# Development of a Biotechnology Platform for the Fast-Growing Cyanobacterium *Synechococcus* sp. PCC 11901

**DOI:** 10.3390/biom12070872

**Published:** 2022-06-23

**Authors:** Lauren A. Mills, José Ángel Moreno-Cabezuelo, Artur Włodarczyk, Angelo J. Victoria, Rebeca Mejías, Anja Nenninger, Simon Moxon, Paolo Bombelli, Tiago T. Selão, Alistair J. McCormick, David J. Lea-Smith

**Affiliations:** 1 School of Biological Sciences, University of East Anglia, Norwich Research Park, Norwich NR4 7TJ, UK; l.mills@uea.ac.uk (L.A.M.); bb2mocaj@uco.es (J.Á.M.-C.); rmejias@us.es (R.M.); s.moxon@uea.ac.uk (S.M.); 2 Bondi Bio Pty Ltd., c/o Climate Change Cluster, University of Technology Sydney, 745 Harris Street, Ultimo, NSW 2007, Australia; artur.wlodarczyk@bondi.bio; 3 SynthSys and Institute of Molecular Plant Sciences, School of Biological Sciences, University of Edinburgh, Edinburgh EH9 3BF, UK; a.j.victoria@sms.ed.ac.uk (A.J.V.); anja.nenninger@ed.ac.uk (A.N.); alistair.mccormick@ed.ac.uk (A.J.M.); 4 Department of Biochemistry, University of Cambridge, Cambridge CB2 1QW, UK; pb346@cam.ac.uk; 5 Department of Chemical and Environmental Engineering, University of Nottingham, Nottingham NG7 2RD, UK; tiago.selao@nottingham.ac.uk

**Keywords:** *Synechococcus* sp. PCC 11901, CodA selection, SacB selection, vitamin B12, photosynthesis, photoinhibition, comparative genomics, cellular metabolism

## Abstract

*Synechococcus* sp. PCC 11901 reportedly demonstrates the highest, most sustained growth of any known cyanobacterium under optimized conditions. Due to its recent discovery, our knowledge of its biology, including the factors underlying sustained, fast growth, is limited. Furthermore, tools specific for genetic manipulation of PCC 11901 are not established. Here, we demonstrate that PCC 11901 shows faster growth than other model cyanobacteria, including the fast-growing species *Synechococcus* *elongatus* UTEX 2973, under optimal growth conditions for UTEX 2973. Comparative genomics between PCC 11901 and *Synechocystis* sp. PCC 6803 reveal conservation of most metabolic pathways but PCC 11901 has a simplified electron transport chain and reduced light harvesting complex. This may underlie its superior light use, reduced photoinhibition, and higher photosynthetic and respiratory rates. To aid biotechnology applications, we developed a vitamin B_12_ auxotrophic mutant but were unable to generate unmarked knockouts using two negative selectable markers, suggesting that recombinase- or CRISPR-based approaches may be required for repeated genetic manipulation. Overall, this study establishes PCC 11901 as one of the most promising species currently available for cyanobacterial biotechnology and provides a useful set of bioinformatics tools and strains for advancing this field, in addition to insights into the factors underlying its fast growth phenotype.

## 1. Introduction

Developing innovative carbon-neutral technologies to substitute for fossil fuel-derived synthetic routes for bulk chemicals and high-value materials production is one of the great challenges of the 21st Century. Cyanobacteria (i.e., prokaryotes that perform oxygenic photosynthesis) are potential renewable biotechnology platforms due to their ability to convert CO_2_ into valuable industrial and pharmaceutical commodities using energy derived from sunlight [1,2]. Moreover, cyanobacterial biomass and bioproduct synthesis does not require arable land, avoiding competition with food production. It also uses minimal nutrients and, for species that can be cultured in seawater, avoids the use of limited freshwater supplies. However, commercialization is dependent on genetically engineering fast-growing strains with high biomass and compound production.

The most widely used model cyanobacterial species are the fresh water species *Synechocystis* sp. PCC 6803 (PCC 6803) and *Synechococcus elongatus* PCC 7942 (PCC 7942), and the marine species *Synechococcus* sp. PCC 7002 (PCC 7002), which have doubling times of approximately 6.6, 4.1 and 4 h [3], respectively. In comparison, heterotrophic species used in industry, such as *Escherichia coli* and *Saccharomyces cerevisiae*, have doubling times of approximately 20 and 90 min, respectively [4,5]. In recent years, several newly discovered species with faster doubling times similar to *S. cerevisiae* have been described including *Synechococcus elongatus* UTEX 2973 (UTEX 2973) [3], *Synechococcus elongatus* PCC 11801 [6] and *Synechococcus* sp. PCC 11901 (PCC 11901) [7]. PCC 11901 reportedly maintains fast growth over extended periods and achieves higher biomass accumulation than PCC 6803, PCC 7942, UTEX 2973, and the closely related species, PCC 7002, when cultured at 30 °C and 38 °C [7]. In comparison, two separate studies showed that growth of UTEX 2973 was slower than PCC 6803 and PCC 7942 when cultured at 30 °C [7,8]. As with other model cyanobacteria, PCC 11901 is naturally transformable but has the added advantage of maintaining a fast growth phenotype over a wide temperature range and can grow in media with similar salinity to seawater, suggesting it may be suitable for outdoor cultivation.

Although PCC 11901 demonstrates many characteristics that make it potentially useful for biotechnology, further testing and adaptation of this species is required to determine whether it is suitable for commercial applications. One disadvantage of PCC 11901 is the requirement of cobalamin (vitamin B_12_) for optimal growth [7]. As is the case for other marine cyanobacteria [9], PCC 11901 has a vitamin B_12_-dependent methionine synthase (MetH) and the initial isolate was shown to be an auxotroph for this cofactor. While a vitamin B_12_-independent methionine synthase gene (*metE*) is present in one of the endogenous plasmids, it seemed to be unable to support growth of the initial isolate in the absence of vitamin B_12_ supplementation [7]. Adding vitamin B_12_ to large-scale cultures would increase the cost of industrial production, which may render production of lower value chemicals commercially non-viable. A further disadvantage is that our understanding of the metabolism and photosynthetic properties of PCC 11901 is limited. This knowledge is required to perform metabolic modeling and flux analysis and may also provide insight into the physiological features underlying the fast, sustained growth phenotype of PCC 11901 [10]. Moreover, a recent analysis suggests that factors other than fast growth are also critical for high phototrophic productivity, including the rate of light absorption, photosynthetic efficiency, and the conversion rate of photons to biomass [11]. Finally, although genetic manipulation of PCC 11901 has been demonstrated, a system for generating unmarked mutants at different chromosomal locations in this species has not been developed. Unmarked mutants are generated via insertion of an antibiotic resistance cassette into the target site, followed by subsequent removal of this cassette using a negative selectable marker. Several negative selectable markers are currently available, including the SacB and CodA systems. SacB confers sensitivity to cells cultured on agar plates with 5–10% sucrose, while CodA confers sensitivity to 5-fluorocytosine (5-FC) [12]. The advantages of this method are that unmarked mutants can be repeatedly genetically manipulated, a prerequisite for development of industrial strains [13]. The absence of genes encoding antibiotic resistance cassettes is also desirable in strains that may be potentially cultured outdoors, avoiding the possibility that they may be transferred to environmental species.

In this study, we demonstrate that PCC 11901 displays the fastest, most sustained growth when compared to a range of model cyanobacterial species, even under the optimal growth conditions for UTEX 2973. Moreover, we show that the fast growth phenotype of PCC 11901 is linked to lower photoinhibition, higher photosynthetic rates, and higher light use compared to other model cyanobacteria. Via comprehensive analysis of central metabolism of PCC 11901, we demonstrate that most pathways are conserved between this species and PCC 6803. Finally, a vitamin B_12_-independent strain was developed, which should lower the cost of large-scale cultivation. However, repeated attempts to generate unmarked mutants using two different negative selectable markers were unsuccessful, suggesting that alternate strategies will have to be pursued for repeated genetic manipulation.

## 2. Materials and Methods

### 2.1. Strain and Culture Conditions

PCC 6803 [14], UTEX 2973 (obtained from the UTEX culture collection of algae), UTEX 2973 (a kind gift from the Pakrasi laboratory at the University of Washington in St Louis) and PCC 7942 (PCC culture collection) were maintained on BG11 agar plates. PCC 11901 was maintained on AD7 plates as described by Włodarczyk et al. [7] while the PCC 11901 B_12_-independent strains (B12_ind_) were grown on AD7 plates not supplemented with vitamin B_12_. All plates were grown at 30 °C under 40 µmol photons m^−2^ s^−1^ light.

Liquid cultures used as starter cultures for growth experiments and genetic manipulation were grown in their corresponding liquid medium in 50 mL volumes in 100 mL conical flasks at 30 °C under continuous 40 µmol photons m^−2^ s^−1^ warm white LED light in an Algaetron growth chamber (Photon Systems Instruments) and shaken at 120 rpm.

### 2.2. Multicultivator MC-1000 Growth Conditions

To determine growth rates, starter cultures were used to inoculate 80 mL cultures in cylindrical cultivation tubes with a diameter of 30 mm to an OD_750nm_ of ~0.1. Tubes were placed into a MC-1000 multicultivator bioreactor (Photon Systems Instruments) and grown at 38 °C under 125 µmol photons m^−2^ s^−1^ warm white LED light and bubbling with air/5% CO_2_. After 24 h of growth, the light intensity increased to 500 µmol photons m^−2^ s^−1^. After a further 24 h of growth, cultures were diluted to an OD_750nm_ of ~0.1 to continue log phase growth and the light intensity increased to 900 µmol photons m^−2^ s^−1^. The cultures were left for approximately 16 h at 900 µmol photons m^−2^ s^−1^ to adapt to the new conditions of the bioreactor. Samples were then diluted to an OD_750nm_ of 0.25 to start the growth experiment. All data were collected via extracting samples and quantification using a Jenway Genova spectrophotometer at 750 nm. Parameters for the growth experiments were adapted from Ungerer et al. [15]. After 72 h of growth at 900 µmol photons m^−2^ s^−1^ and 38 °C, 5 mL of culture was harvested from each strain to quantify biomass. Cells were centrifuged at 5000× *g* and washed twice with sterile H_2_O. Whatman 0.7 μM GF/B Glass Microfibre Filters of 70 mm diameter were measured on a microbalance three times to obtain an average filter weight. The washed cells were then added to the filters, and left to dry for 24 h at 60 °C. The filters were then weighed again three times to obtain the average weight of the filter plus the biomass for each species. A student’s unpaired *t* test was used to compare growth and biomass accumulation of PCC 11901 against the other cyanobacterial species, *p* < 0.05 being considered statistically significant.

### 2.3. Bioinformatics Analysis and Generation of a Metabolic and Electron Transport Map for PCC 11901

Comparative proteome analysis for PCC 11901 was performed against the proteome of PCC 6803 using the National Centre for Biotechnology Information’s (NCBI) basic local alignment search tool (BLAST) [16]. To compare against the well-documented PCC 6803 species, the input data were based on the findings of a modified version of Table S3 from Baers et al. [17]. This formed the foundation for the input data for the following data analysis. A second input file was then taken from the tables found in Mills et al. [18] which group PCC 6803’s proteome into metabolic categories.

PCC 11901 proteome release CP040360.1 was downloaded from NCBI GenBank and a BLAST database was created using BLAST+ v.2.11.0, using the default parameters. The protein sequences associated with each PCC 6803 Uniprot ID were searched against the PCC 11901 database using the following parameters: output format was set to -outfmt 7 with an e-value set to maximum 1. The e-value was kept quite liberal for future downstream processing. All PCC 11901 sequences without a significant BLAST hit were considered to be being unique to the PCC 11901 proteome. The computer coding for this project can be found at https://github.com/laurenmills300/PCC6803_compare_proteome (assessed on 14 May 2022).

### 2.4. Oxygen Electrode Measurements

Cultures were grown in a MC-1000 multicultivator bioreactor at 30 °C under 125 µmol photons m^−2^ s^−1^ warm white LED light and bubbling with air/5% CO_2_. UTEX 2973 obtained from the UTEX culture collection of algae was used for these measurements. Cells were harvested during log phase at an OD_750nm_ of ~1 and diluted to 1 nmol Chl^−1^ mL^−1^ for analysis of photoinhibition or 4 nmol Chl^−1^ mL^−1^ for photosynthesis and respiration measurements. The amount of chlorophyll in each species was determined by subtracting the 750 nm OD value from the 680 nm value and multiplying the total by the slope of the regression line, as previously performed in Lea-Smith et al. for PCC 6803 [19] (Figure S1). For each species, there was a strong correlation in determining chlorophyll concentration between this method and the well-established chlorophyll quantification protocol described in Porra et al. [20]. All oxygen measurements were carried out using the appropriate media for each strain, and measurements were taken using the Oxytherm+ photosynthesis Clark-electrode (Hansatech Instruments, Kings Lynn, UK). For photoinhibition experiments, samples were kept at 30 °C in the oxygen electrode chamber in the dark for a 10 min period before being subjected to 2000 µmol photons m^−2^ s^−1^ of light. Photoinhibition experiments were conducted either in the absence of presence of lincomycin (200 µg mL^−1^). For photosynthesis measurements the cultures were first dark equilibrated for a 10 min dark period, before being subject to increasing levels of light for 10 min at 10, 25, 50, 150, 350, 900, and 2000 µmol photons m^−2^ s^−1^, respectively. A 10 min dark period preceded each increase in light intensity. NaHCO_3_ was added to each sample to a final concentration of 10 μM. A total of 4–10 biological replicates was tested for each experiment. A student’s unpaired *t* test was used for comparison of PCC 11901 versus the other cyanobacterial species, *p* < 0.05 being considered statistically significant.

### 2.5. Development of Vitamin B_12_-Independent PCC 11901 Strains

To generate a vitamin B_12_-independent PCC 11901 strain, a wild-type culture was grown to OD_750nm_ ~ 2 in AD7 medium, in air/1% CO_2_ and 660 µmol photons m^−2^ s^−1^ continuous light intensity, as previously described [7]. The culture was centrifuged (6000× *g*, 10 min, room temperature) and cells were washed twice with AD7 without B_12_ supplementation (AD7 B_12_^−^) before being serially diluted (from 10^−3^ to 10^−6^) and 100 µL spread on AD7 B_12_^−^ agar plates. Plates were incubated under air/1% CO_2_ and 300 µmol photons m^−2^ s^−1^ continuous light until colonies appeared after approximately two weeks. The five largest colonies across all plates were re-streaked at least six times on AD7 B_12_^−^ plates under the same conditions, until robust growth in AD7 B_12_^−^ agar was consistently obtained. The best growing strain (B12ind_5) was deposited in the UTEX culture collection under strain number UTEX 3154.

Starter cultures for growth experiments were cultured in 50 mL volumes in 100 mL conical flasks at 30 °C in AD7 medium lacking vitamin B_12_ for wild-type and the B_12_-independent mutants and with vitamin B_12_ for a separate wild-type culture. Wild-type and B_12_-independent mutants were then cultured in the MC-1000 multicultivator at 38 °C with air/5% CO_2_, in AD7 B_12_^−^ medium, under continuous warm white light of 125 µmol photons m^−2^ s^−1^ before an aliquot was transferred to a new culture grown at 300 µmol photons m^−2^ s^−1^. This was diluted to OD_750nm_ = 0.25 and growth determine approximately every 24 h. A separate wild-type PCC 11901 culture was grown under the same conditions in the presence of vitamin B_12_.

### 2.6. Cloning of the metE Upstream Region and Generation of B_12_-Independent Strains by Targeted Mutagenesis

A 1000 bp region consisting of the first 500 bp of the coding sequence of *metE* (GenBank accession number: QCS51047.1) and the 500 bp region upstream of the metE start codon was amplified from both WT PCC 11901 and the B_12_ind_5 B_12_-independent strain using primers ICA_11901metE_pUC19_F and ICA_11901metE_pUC19R (Table S1). This fragment was inserted into linearized pUC19 generated using primers ICA_pUC19_11901metE_F and ICA_pUC19_11901metE_R and cloned via *E. coli* mediated assembly [21], resulting in plasmids pTS011 and pTS012, respectively (Figure S2). Inserts were Sanger sequenced using primers metE_seq_1, metE_seq_2 and metE_seq_3. Plasmid pTS012 was used to transform wild-type PCC 11901. Briefly, 2 µg plasmid were added to 2 mL of a culture at OD_750nm_ = 0.5 and incubated overnight at 30 °C with 1.5% air/CO_2_, under continuous warm white light of 300 µmol photons m^−2^ s^−1^, with shaking at 150 rpm. The following day the culture was centrifuged 5 min at 5000× *g* and the pellet washed with 1 mL AD7 B_12_^−^ medium before plating on AD7 B_12_^−^ agar medium plates. These were incubated under the same conditions until colonies became apparent. The five largest colonies across all plates were re-streaked at least six times on AD7 B_12_^−^ plates under the same conditions, until robust growth in AD7 B_12_^−^ agar was consistently obtained. Growth of a B_12_-independent mutants in B_12_^−^ liquid medium was then assessed as described above. A student’s unpaired *t* test was used for comparison of wild-type + vitamin B_12_ versus the other strains and wild-type—vitamin B_12_, *p* < 0.05 being considered statistically significant.

### 2.7. Generation of PCC 11901 Unmarked Deletion Mutants

PCC 11901 knockouts were constructed according to a protocol similar to that used in PCC 6803 and outlined in Lea-Smith et al. [13], except plasmids were constructed using the CyanoGate system [8]. All primers used for cloning and mutant verification are listed in Table S1 and plasmids are listed in Table S2.

Assembly of the plasmid for generating marked mutants was performed using a standard Golden Gate one-pot digestion/ligation reaction [22]. The *codA* gene in each cassette is under control of the promoter J23101 [8]. A 20 µL reaction was prepared by adding 2 µL of Ligase Buffer (10×); 2 µL of bovine serum albumin (BSA; 1 mg/mL); 1.5 µL of *BsaI* (NEB); 0.5 µL of T4 ligase (NEB); 40 ng of the pUC19 backbone vector; 40 ng of the *codA*/*kanR* (conferring kanamycin resistance), *codA*/*specR* (conferring spectinomycin resistance) or *sacB*/*specR* cassette; 10 ng of the PCR product of the left-flanking region; 10 ng of the PCR product of the PCR right-flanking region. The reaction was then cycled in a programmed thermocycler for 35 cycles, first at 37 °C for 5 min, then 16 °C for 5 min, with a final hold at 60 °C for 5 min to inactivate the enzymes. The assembly mixes were then transformed into NEBStable *E. coli* (New England Biolabs). To confirm correct assembly, colony PCR was performed with primers Km_LF_QC, pUC19_LR_QC, pUC19_RF_QC and Cassette_RR_QC and either a double digest involving the restriction enzymes SacI-HF (NEB) and ScaI-HF (NEB), or a single digest with SacI-HF was used, followed by sequencing with primers pUC19_RF_QC and pUC19_LR_QC.

Assembly of the plasmid for generating unmarked mutants was performed using a similar method as that described above; however, the restriction enzyme used in the Golden Gate assembly was *BpiI* (Thermo Fisher). A 20 µL reaction was generated by adding 2 µL of Ligase Buffer (10×); 2 µL of BSA (1 mg/mL); 1.5 µL of *BpiI*; 0.5 µL of T4 ligase (NEB); 40 ng of the marked plasmid; 40 ng of the pUC19KL CyanoGate linker. To confirm correct assembly, either a double digest involving the restriction enzymes *SacI*-HF (NEB) and *ScaI*-HF (NEB), or a single digest with *SacI*-HF was used.

Multiple methods were used to generate marked mutants. To generate marked mutants of *desB*, approximately 1 µg of the marked plasmid was mixed with PCC 11901 cells for 24 h in liquid AD7 media or BG11 medium before plating onto AD7 or BG11 agar plates, respectively, supplemented with 25 ug/mL spectinomycin. In addition, the marked plasmid was mixed with PCC 11901 cells in BG11 medium for either 4 or 24 h prior to plating on either AD7 or BG11 agar plates. Cells that were plated after 4 h were incubated for 24 h, with an additional 3 mL of agar containing spectinomycin added to the surface of the plate, followed by further incubation for approximately 1 week. To generate marked mutants of *ctaC1D1E1* and *ctaCII*, approximately 1 µg of the marked plasmid was mixed with PCC 11901 cells for 4 h in liquid AD7 media, followed by incubation on AD7 agar plates for approximately 24 h. An additional 3 mL of agar containing kanamycin was added to the surface of the plate followed by further incubation for approximately 1–2 weeks. Single colonies were re-streaked onto AD7 plates containing either 25 µg/mL spectinomycin, 50 µg/mL kanamycin or 100 µg/mL kanamycin. Segregation of all strains was confirmed by PCR using the primers DesBfor/DesBrev, COXfor/Coxrev, and ARTOfor/ARTOrev, which flank the deleted region of the respective genes.

To test the susceptibility of wild-type and marked strains to 5-FC, starting cultures were grown to OD_750nm_ = 3 and 20 μL of cells of different dilutions were plated on agar containing concentrations of 5-FC up to 1 mg/mL. Plates were grown for five days. To attempt to remove the *codA/KanR* cassette or *codA/SpecR* cassette, multiple methods were trialed. In the first, the mutant line was transformed with 1 µg of the respective markerless constructs. Following incubation in AD7 liquid media for four days and agar plates containing 5-FC for a further 1–2 weeks, transformants were patched onto AD7 plates with kanamycin (100 ug/mL) or spectinomycin (25 ug/mL), and 5-FC plates (0.5 mg/mL). Mutants which were 5-FC resistant but kanamycin or spectinomycin sensitive were checked using the primers DesBfor/DesBrev, COXfor/Coxrev and ARTOfor/ARTOrev, which flank the deleted region of the respective genes. In the second method, unmarking was performed by taking 2 mL of OD_750nm_ = 0.5 marked desB:codA-SpR mutants and washing using antibiotic-free AD7 medium thrice to remove any spectinomycin. Afterwards, 1 µg of unmarking plasmid were mixed with the washed cells and incubated for four days in a shaking incubator before plating onto AD7 agar supplemented with 250 ug/mL 5-FC to select for unmarked mutants. Putative unmarked colonies were then streaked onto increasing concentrations of 5-FC up to 1 mg/mL to encourage segregation. Streaks that survived and grew on 5-FC supplemented agar were screened using the primers DesBfor/DesBrev to check for the presence of the unmarked band and the loss of the marked band.

## 3. Results

### 3.1. Synechococcus sp. PCC 11901 Is the Fastest Growing Species under High Light at 38 °C

**Figure 1:**
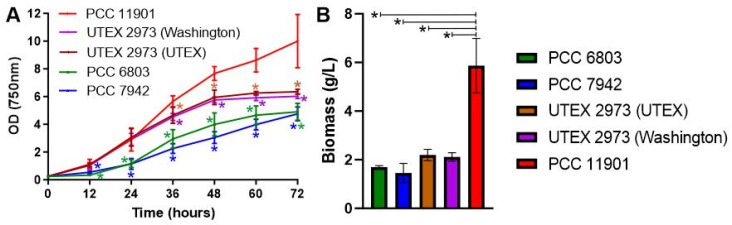
**(A) Growth and (B) biomass accumulation of cyanobacterial species.** Strains were cultured at 38 °C under 900 µmol photons m^−2^ s^−1^ continuous light intensity and with direct bubbling of air/5% CO_2_. Error bars indicate SD. Asterisks indicate significant differences between PCC 11901 and the other cyanobacterial species (*p* < 0.05).

In a recently published comparison, PCC 11901 demonstrated faster growth and higher biomass accumulation than PCC 7002, UTEX 2973, PCC 7942 and PCC 6803 at 30 °C under 750 µmol photons m^−2^ s^−1^ continuous light intensity [7]. PCC 11901 also outperformed PCC 7002 and UTEX 2973 at a higher temperature of 38 °C and continuous light of 300 and 660 µmol photons m^−2^ s^−1^ [7]. However, in each growth experiment, 25 mL cultures were grown in 125 mL extra deep baffled flasks (Corning) in a chamber sparged with air/1% CO_2_. This differs from the growth conditions in which UTEX 2973 was demonstrated to have optimal growth, specifically at 38 °C under 900 µmol photons m^−2^ s^−1^ continuous light and with direct bubbling of air/5% CO_2_, in a MC-1000 multicultivator [15]. We therefore cultured PCC 11901, UTEX 2973, PCC 7942 and PCC 6803 under these exact growth conditions to determine whether PCC 11901 still outperformed the other strains. Two different UTEX 2973 strains, one obtained from the UTEX collection, the other from the laboratory that performed the initial growth studies, were tested. This was to ensure that any possible differences we observed from the initial study were not due to mutations in UTEX 2973 strains. Under these growth conditions, both UTEX 2973 strains demonstrated fast growth in the first 24 h, similar to previous studies [3,15] and comparable to growth of PCC 11901 (Figure 1A). However, after 36 h, growth of both UTEX 2973 strains was significantly slower than PCC 11901 and by 48 h had entered stationary phase at an OD_750nm_ = ~6. In contrast, after 72 h PCC 11901 was OD_750nm_ = ~10 and growth was still in exponential phase. As demonstrated previously, growth of UTEX 2973 was faster than PCC 7942 and PCC 6803 [3]. Moreover, biomass accumulation was significantly higher in PCC 11901 than the other species after 72 h (Figure 1B). However, the OD_750nm_/biomass (g/L) ratio was 1.7 in PCC 11901, compared to 2.88 in PCC 6803, 3.26 in PCC 7942, 2.9 in UTEX 2973 (UTEX) and 2.85 in UTEX 2973 (Washington), suggesting that measuring growth by spectrophotometry alone may not be a good method of determining growth of PCC 11901. This could be due to an increase in size of PCC 11901 cells, which has been reported to occur at later stages of growth [7] or to the other species entering or being in stationary phase when biomass was quantified. Overall, PCC 11901 demonstrated the fastest sustained growth and highest biomass accumulation of the species tested under optimal UTEX 2973 growth conditions, further emphasizing its potential for biotechnology applications.

### 3.2. Metabolic Pathways Are Highly Conserved between Synechococcus sp. PCC 11901 and Synechocystis sp. PCC 6803

The biological traits of PCC 11901 leading to faster growth have not been identified. To determine whether differences in metabolism are potentially responsible for differing growth rates and to develop a metabolic network which would aid future modeling, flux balance analysis, and metabolic engineering, we performed a comparative genomics analysis of PCC 6803 versus PCC 11901 using the BLAST local databases (Table S3). To evaluate the metabolic and transport capacity of PCC 11901 we compared it to the pathways outlined in a recent analysis of PCC 6803 [18].

The PCC 11901 genome contains genes encoding most of the enzymes required for central metabolism with a few exceptions (Figure S3). RpiB, a probable ribose phosphate isomerase is absent. However, a homologue encoding its isoenzyme, RpiA, is present in PCC 11901 and is likely sufficient for catalyzing this reaction in the oxidative pentose phosphate and Calvin-Benson-Bassham pathways. Fba1 is also absent but is likely compensated for by expression of Fba2. AckA, encoding acetate kinase, required for production of acetate via the Pta/AckA pathway is absent, although the homologue for Acs, required for an alternative acetate biosynthesis pathway, is present. Poor homology of PCC 11901 proteins is demonstrated to several enzymes involved in the oxidative pentose phosphate pathway (Transaldolase (Tal): e-value = 8.89e-08), branched glycogen catabolism (Isoamylase (GlgX1 and GlgX2): e-value = 2.54e-09 and 1.48e-12, respectively) and the Tricarboxylic Acid Cycle (Isocitrate dehydrogenase (NADP+; Icd): e-value = 3.69e-15). However, a putative NADP+ dependent isocitrate dehydrogenase is encoded by WP_138073772.1 in the PCC 11901 genome and likely performs this last function. There are four putative phosphoglycolate phosphatase enzymes in PCC 6803, encoded by *slr0458*, *slr0586*, *sll1349*, and *slr1762* [23], but only two of these (Slr0458 and slr1762) have homologues in PCC 11901 with strong similarity (WP_138072486.1; e-value = 2e-62 and WP_030006614.1; 2e-55, respectively). The last enzyme in the photorespiration pathway, GlyK, is also absent in PCC 11901, which is also the case in PCC 7002. A comparison to PCC 7002 also demonstrated poor similarity of Tal (1e-07) and Icd (4e-15) with PCC 6803 homologues. Genes encoding proteins involved in polyhydroxybutyate biosynthesis are not present, as previously reported [7].

The majority of proteins involved in metabolism and degradation of nucleotide sugars and sugar osmolytes are present (Figure S4), except for genes encoding the proteins involved in sucrose degradation (Inv, Glk (sll0593)) and possibly FrkA (e-value = 3.4e-19). Homologues to RfbC, the third enzyme in the TDP-rhamnose biosynthetic pathway, are not present in PCC 11901 or PCC 7002 and it is not known whether PCC 11901 synthesizes rhamnose.

Pathways involved in metabolism of amino acids, cyanophycin, glutathione, and iron-sulfur clusters are conserved between both species (Figure S5), except for *gltD*, encoding the small subunit of the NADH-dependent glutamate synthase. Likewise, pathways involved in nucleotide metabolism are highly conserved between both species (Figure S6) with the exception of the poorly characterized thymidylate synthase ThyX. The Dgt and CodA nucleotide salvage pathways [18] are absent in PCC 11901. Metabolism of vitamins and co-factors is similar between both species (Figure S7), except for the absence of homologues encoding NadV and NadM, which convert nicotinamide to NAD^+^.

Compared to PCC 6803, cell wall metabolism in PCC 11901 is similar except it lacks one of the desaturases encoded by PCC 6803 (DesD), produces hydrocarbons via the Ols pathway, instead of via FAD/FAR, which is similar to *Synechococcus* sp. PCC 7002 (Figure S8) [24], and does not contain genes encoding penicillin binding proteins 6 and 7. Metabolism of isoprenoids, quinols, carotenoids, chlorophyll, and phycobilin is also similar (Figures S9 and S10). However, PCC 11901 does not contain genes encoding the hopene biosynthetic pathway and as previously reported, it lacks multiple genes in both the anaerobic and aerobic pseudocobalamin (vitamin B_12_) pathway [7].

Genes encoding subunits of many transporters are also absent in the PCC 11901 genome (Figure S11). These include transporters importing basic amino acids (Bgt complex) and glutamate (Gtr complex, GltS), suggesting that only the Nat complex can import amino acids. Potassium transport systems are greatly reduced in PCC 11901, which lack the Kdp K^+^ import complex and the thylakoid membrane localized SynK protein, required in PCC 6803 for optimal photosynthesis [25]. Subunits of the magnesium (MgtC) and the sulfate (SbpA) import complexes and the Nrs complex, exporting nickel, zinc, and copper (Figure S9) are also absent in PCC 11901. GlcP, involved in glucose uptake is absent in PCC 11901, possibly explaining the inability of this species to grow heterotrophically on glucose [7].

Genes not encoding proteins involved in central metabolism, electron transport and light harvesting, or with unknown function, but conserved in PCC 6803 and PCC 11901, are listed in Table S4. We did not investigate pathways absent in PCC 6803 that may be present in PCC 11901 (Table S5). There are 353 PCC 11901 genes which are distinctly different from any homologues in PCC 6803 and it is possible that some may encode for enzymes synthesizing metabolites that could play a role in enhancing growth in this organism.

### 3.3. Electron Transport and Light Harvesting Is Streamlined in PCC 11901 Compared to PCC 6803

**Figure 2:**
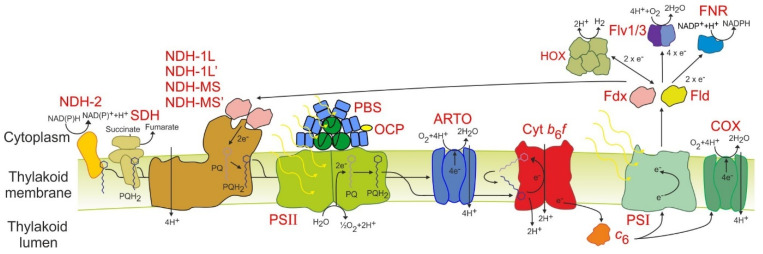
**Schematic diagram of the proposed PCC 11901 electron transport chain network.** NDH-2: NAD(P)H dehydrogenase 2; SDH: Succinate dehydrogenase; NDH-1L/1L’/MS/MS’: NAD(P)H dehydrogenase 1 complexes; PBS: Phycobilisome; OCP: Orange Carotenoid Protein; PSII: Photosystem II; PQ: plastoquinone; PQH2: plastoquinol; ARTO: Alternative respiratory terminal oxidase; Cyt *b*_6_*f*: Cytochrome *b*_6_*f*; Cyt *c*_6_: cytochrome *c*_6_; PSI: Photosystem I; Fdx: ferredoxin; COX: cytochrome-*c* oxidase; Fld: Flavodoxin; FNR: ferredoxin-NADP+-reductase; Flv1/3: Flavodiiron 1/3; HOX: Bidirectional hydrogenase.

Next we compared the differences between PCC 11901 and PCC 6803 in cellular processes, including electron transport and light harvesting (Table S6; Figure 2) [26]. All subunits of the three main complexes, photosystem II and I, and cytochrome *b*_6_*f*, are present. Similar to PCC 7002, PCC 11901 encodes cytochrome *c*_6_ (*c*_553_) but plastocyanin is not present [27]. Both electron carriers, ferredoxin (Fdx) and flavodoxin (Fld), in addition to ferredoxin-NADP+ reductase, are present. Only two flavodiiron proteins are encoded in the PCC 11901 genome and these demonstrate the closest similarity to PCC 6803 Flv1 and Flv3, suggesting that only the Flv1/Flv3 complex is present in this species. All the subunits of the hydrogenase are also present. Other putative electron transport proteins, including the enigmatic CytM and Pgr5 proteins, are present [28,29]. Ten putative ferredoxins are also potentially encoded in the PCC 11901 genome, of which only Fed2, involved in iron response [30], was characterized.

Subunits specific to each of the four NAD(P)H dehydrogenase-like complexes (NDH-1L, NDH-1L’, NDH-MS, NDH-MS’) are present in PCC 11901 [31]. All subunits of succinate dehydrogenase (SDH) are present, including the recently discovered putative third subunit encoded by Slr0201 [32]. Two NAD(P)H dehydrogenase II proteins (NdbA, NdbB) are also present but there is no homologue to NdbC. Of the terminal oxidases, only genes encoding subunits of the quinone oxidizing *bo*_3_-type alternative respiratory terminal oxidase (ARTO) and the *aa*_3_-type cytochrome-*c* oxidase complex (COX) are present in the genome. In PCC 7002, deletion of ARTO and COX increased reduction rates of photosystem I, suggesting that both complexes are thylakoid membrane localized [33]. While PCC 6803 incorporates a separate electron transport chain in the plasma membrane, the lack of a third terminal oxidase suggests that this chain may not be present in PCC 11901. However, proteome mapping, as recently performed in PCC 6803 [17], would be required to confirm this.

In terms of light harvesting, the phycobilisome (PBS) has only one CpcC linker protein, suggesting that each rod consists of only two stacked disk-shaped phycocyanin hexamers radiating out of the allophycocyanin core. This PBS structure is likely similar to PCC 7002 but differs from PCC6803, UTEX 2973, and PCC7942, which encode two CpcC linker proteins and have three stacked disk-shaped phycocyanin hexamers per rod. Given that PCC 7002 and PCC 11901 demonstrate faster sustained growth at high cell densities compared to the other three species this suggests that PBSs with two stacked disk-shaped phycocyanin hexamers may be optimal for light harvesting. The orange carotenoid protein is also present.

### 3.4. PCC 11901 Demonstrates Higher Photosynthetic and Respiratory Rates, Lower Photoinhibition and Superior Light Use Compared to Other Model Cyanobacteria

**Figure 3:**
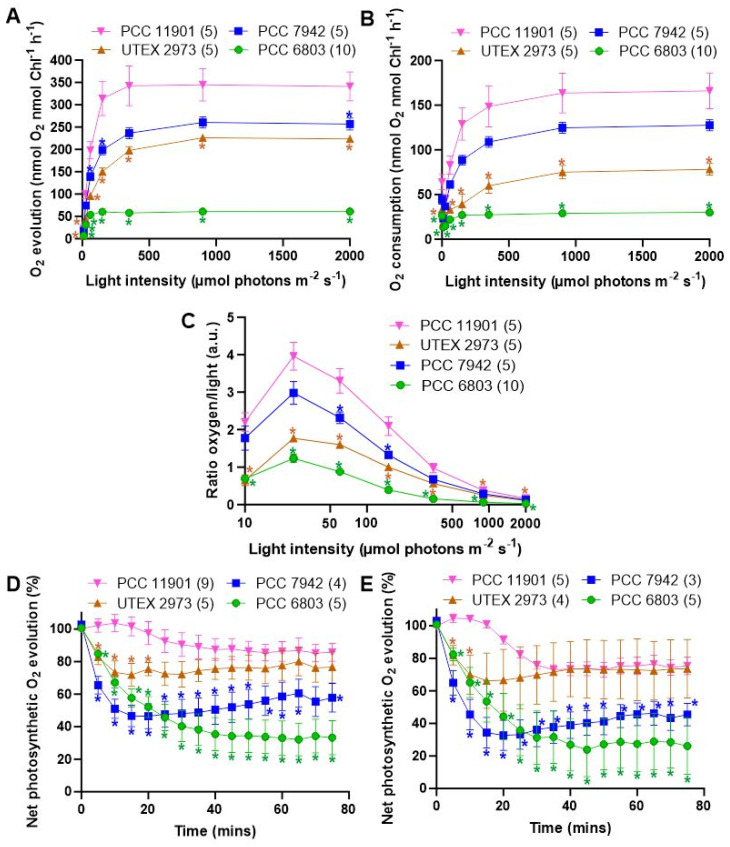
**Characterization of the photosynthetic and respiratory rates, light use and photoinhibition of cyanobacterial species**. (**A**) Oxygen evolution was measured at different light intensities, and (**B**) oxygen consumption was measured following each light period. (**C**) The coefficient of light use was calculated by dividing the net rate of oxygen evolution by the correspondent light photon flux. Photoinhibition was quantified by determining photosynthetic oxygen evolution in the (**D**) absence and (**E**) presence of lincomycin. All results are from three to ten biological replicates (number indicated in brackets after species legend). Errors bars indicate SE. Color-coded asterisks indicate significant differences between PCC 11901 and the other cyanobacterial species (*p* < 0.05).

We then sought to determine whether the photosynthetic properties of PCC 11901 may play a role in the higher growth rates observed in this species. This was performed with cultures grown at 30 °C under 125 µmol photons m^−2^ s^−1^ warm white LED light and bubbling with air/5% CO_2_ to test species under the high carbon saturation conditions optimal for PCC 11901. A lower light intensity was selected to avoid stress on the cells while cells were cultured at 30 °C to allow direct comparison to previous experiments performed on PCC 6803 [34,35], and because this is the optimal temperature for PCC 6803 and PCC 7942. UTEX 2973 also demonstrates similar growth to PCC 11901 under the first 48 h of growth at 30 °C [7], allowing a direct comparison between this species and PCC 11901 during this period. Photosynthetic rates were measured at different light intensities to generate a saturation curve, with a dark period preceding each increase in light intensity (Figure 3A). In such curves, the net rate of oxygen evolution levels off as saturating light intensity is approached, with the maximum rate of oxygen evolution designated as P_max_. The P_max_ of PCC 11901 was 32%, 52% and 566% higher than PCC 7942, UTEX 2973 and PCC 6803, respectively. The rate of oxygen depletion in the dark after each period of illumination (i.e., cellular respiration) can also be plotted as a function of the light intensity prior to the dark period, giving the respiration curve (Figure 3B). The maximum rate of oxygen depletion was measured after the highest light intensity. The maximum rate of oxygen depletion of PCC 11901 was 30%, 212% and 552% higher than PCC 7942, UTEX 2973 and PCC 6803, respectively.

PCC1101 demonstrated superior light use compared to the other species. When the net rate of oxygen evolution is divided by the corresponding light photon flux, the coefficient of light use (a.u.) can be calculated. PCC 11901 demonstrated superior light use to PCC 7942 at 50 and 100 µmol photons m^−2^ s^−1^ light and PCC 6803 and UTEX 2973 at all light intensities (Figure 3C).

To test for photoinhibition, all species were first incubated in the dark for 10 min, followed by constant exposure to constant saturating light of 2000 µmol photons m^−2^ s^−1^ for 75 min in the absence and presence of lincomycin (Figure 3D,E, respectively), during which time oxygen evolution was measured. Photoinhibition, as determined by a decrease in oxygen evolution was lowest in PCC 11901. In the absence of lincomycin the rate of oxygen evolution decreasing to 85.7% ± 5.5%, 76.6% ± 9.7%, 57.8% ± 8.8% and 33.2% ± 10.5% for PCC 11901, UTEX 2973, PCC 7942 and PCC 6803, respectively. Addition of lincomycin, an inhibitor of protein synthesis and thus repair of PSII, resulted in similar levels of photoinhibition between PCC 11901 and UTEX 2973, with the rate of oxygen evolution decreasing to 75.4 ± 5.4% and 73.6% ± 18.3%, respectively. Higher rates of photoinhibition were observed in PCC 7942 and PCC 6803, with the rate of oxygen evolution decreasing to 45.4 ± 6.9% and 26.1% ± 17.4%, respectively.

### 3.5. Generation of a Vitamin B_12_-Independent Synechococcus sp. PCC 11901 Strain

**Figure 4:**
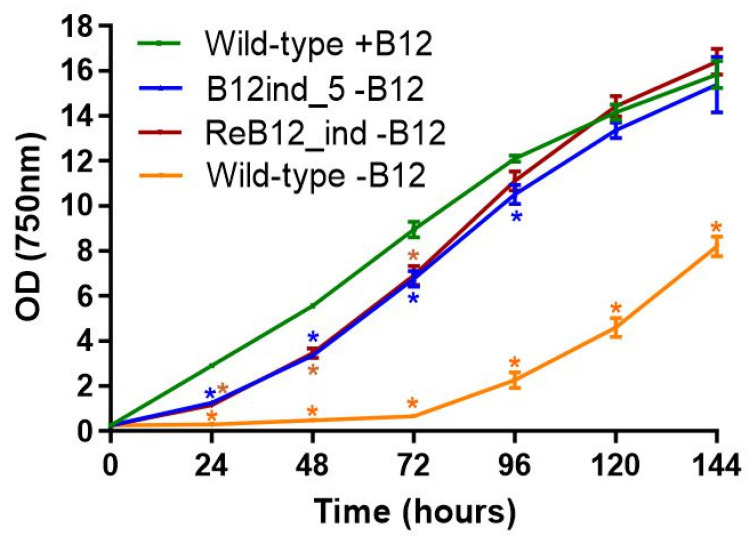
**Growth of wild-type and B_12_-independent mutants in liquid AD7 medium.** Wild-type was cultured in B_12_^−^ and B_12_^+^ medium. Strains were cultured at 38 °C under 300 µmol photons m^−2^ s^−1^ continuous light intensity and with direct bubbling of air/5% CO_2_. Error bars indicate SD. Asterisks indicate significant differences between wild-type +B12 and the other strains and wild-type −B12 (*p* < 0.05).

While PCC 11901 was shown to be a robust strain for biotechnology, its vitamin B_12_ auxotrophy could be a deterrent for many industrial applications, given it increases cultivation costs at scale. We therefore sought to overcome this limitation by isolating spontaneous vitamin B_12_-independent PCC 11901 mutants. A WT PCC 11901 culture was diluted and spread on AD7 B_12_^−^ agar plates, with the five largest colonies across all plates re-streaked at least six times on AD7 B_12_^−^ plates under the same conditions, until robust growth on AD7 B_12_^−^ agar was consistently obtained. The fastest growing strain (B12ind_5) demonstrated initial lower growth than wild-type when cultured in liquid medium but had grown to a similar density by 120 h, suggesting that this strain was viable in the absence of vitamin B_12_ (Figure 4). Sequencing of the B_12_ind_5 upstream region of the *metE* gene revealed two point mutations (TT->AA), 320 bp upstream of the start codon (Figure S12). Plasmid pTS012 was used to transform WT PCC 11901 and the resulting strain (ReB12_ind), selected on B_12_-free AD7 medium, had a similar growth profile to that of the B_12_ind_5 strain (Figure 4). In contrast, wild-type cultured in vitamin B_12_^−^ liquid medium demonstrated far lower growth although after 72 h these cultures began to adapt to vitamin B_12_^−^ conditions.

### 3.6. The CodA and SacB Negative Selectable Markers Were Not Successfully Used in Synechococcus sp. PCC 11901 for Generating Unmarked Mutants

While genetic manipulation via natural transformation of PCC 11901 has been demonstrated [7], including marked gene deletions and chromosomal insertion of gene cassettes, a system for repeated chromosomal unmarked modification has not been developed. This is essential for biotechnology applications since multiple chromosomal modifications will likely be necessary to generate strains of commercial value. Generation of marked mutations in multiple chromosomal locations is limited by the number of available cassettes conferring antibiotic resistance. To generate mutants that can be genetically manipulated repeatedly, we adapted the two-step unmarked mutant protocol previously developed for PCC 6803 [13]. We tested two negative selectable markers, *sacB* and *codA*. *sacB* is commonly used for unmarking in PCC 6803, but is ineffective in PCC 7002, the species most genetically similar to PCC 11901 [13,36]. *codA*, in this cassette under control of the J23101 promoter [8], encodes a cytosine deaminase protein which converts 5-FC to the toxic agent 5-fluorouracil [37].

We targeted *desB*, a neutral site in PCC 7002 [38], using a plasmid in which cassettes containing *sacB* and a gene conferring spectinomycin resistance, was inserted between regions in the chromosome flanking the deletion site. This plasmid was transformed into PCC 11901 using spectinomycin to select for potential marked knockouts. However, despite three separate transformation attempts, no colonies were observed, suggesting that expression of *sacB* is lethal to PCC 11901 even in the absence of sucrose on agar plates. Since PCC 11901 also encodes a putative sucrose biosynthesis pathway (Figure S4), which is typically expressed in cyanobacteria under high-salt conditions [39], we also attempted to generate marked mutants on low-salt BG11 medium plates. Despite trying two different transformation methods in which the incubation time in liquid BG11 medium was altered (4 and 24 h), and the cells plated on either BG11 or AD7 solid medium, no colonies were obtained. Therefore, it seems unlikely that this negative selectable marker can be used to generate unmarked mutants.

**Figure 5:**
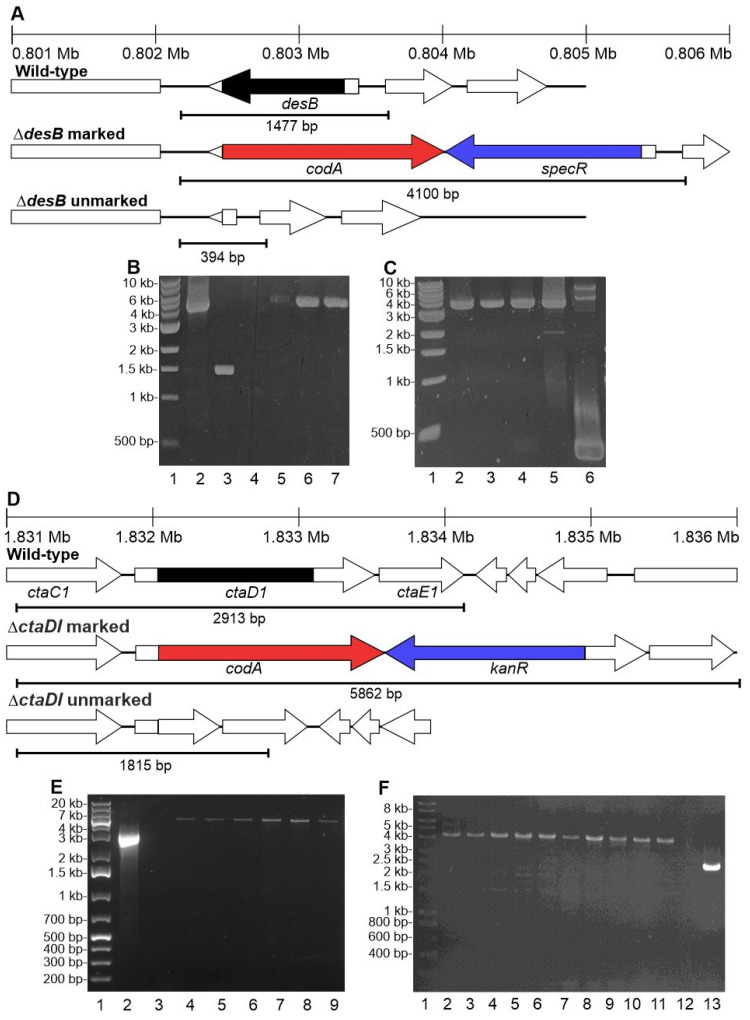

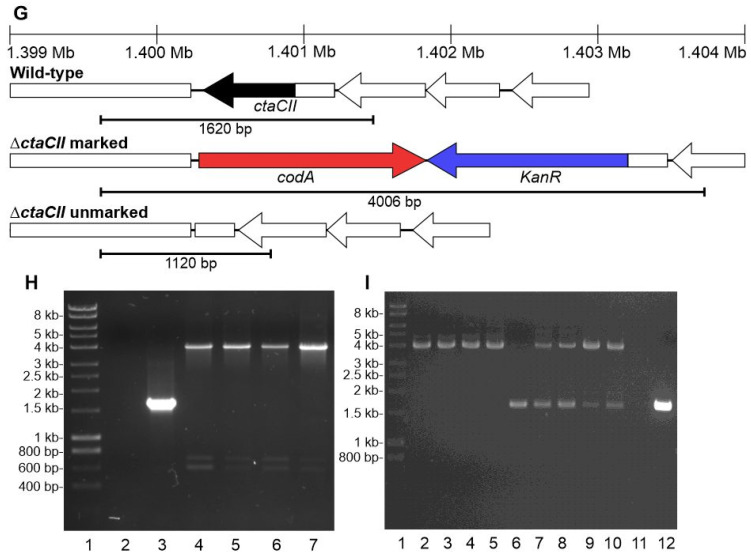
**Generation of marked knockouts and attempted generation of unmarked knockouts in PCC 11901.** DNA ladders are shown in lane 1 in each panel. (**A**) Schematic representations of locus location in the PCC 11901 genome (top) and profiles expected in wild-type and the ∆*desB* marked and unmarked knockouts. (**B**) Amplification of genomic DNA in WT (Lane 3; Expected band size: 1477 bp) and ∆*desB* marked mutants (Lanes 4–7) using DesBfor and DesBrev primers. Positive control is shown in lane 2. (**C**) Amplification of genomic DNA in the *desB* marked mutant (Lanes 5) and putative *desB* unmarked mutants (Lanes 2–4, 6) using DesBfor and DesBrev primers. (**D**) Schematic representations of locus location in the PCC 11901 genome (top) and profiles expected in wild-type and the ∆*ctaDI* marked and unmarked knockouts. (**E**) Amplification of genomic DNA in WT (Lane 2) and ∆*ctaDI* marked mutants (Lanes 4–9) using COXfor and COXrev primers. Negative control (no gDNA) is shown in lane 3. (**F**) Amplification of genomic DNA in putative ∆*ctaDI* unmarked mutants (Lanes 2–11) using COXfor and COXrev primers. Negative control (no gDNA) is shown in lane 12. Amplification of wild-type is shown in lane 13. (**G**) Schematic representations of locus location in the PCC 11901 genome (top) and profiles expected in wild-type and the ∆*ctaCII* marked and unmarked knockouts. (**H**) Amplification of genomic DNA in WT (Lane 3) and ∆*ctaCII* marked mutants (Lanes 4–7) using ARTOfor and ARTOrev primers. Negative control (no gDNA) is shown in lane 2. (**I**) Amplification of genomic DNA in putative ∆*ctaCII* unmarked mutants (Lanes 2–10) using ARTOfor and ARTOrev primers. Negative control (no gDNA) is shown in lane 11. Amplification of wild-type is shown in lane 12.

In contrast, transformation with plasmids in which *codA* replaced *sacB* as the negative selectable marker resulted in approximately 5–40 colonies per microgram of plasmid. Segregation of marked mutants was obtained after two further streaks on 25 μg/mL spectinomycin containing plates (Figure 5B). Marked mutants of *ctaC1D1E1* and *ctaCII*, encoding subunits of cytochrome-*c* oxidase (COX) and the alternative respiratory terminal oxidase (ARTO), respectively, were also generated (Figure 5E,H) using kanamycin for selection. These are the only terminal oxidases in PCC 11901 and were selected because these genes are non-essential in PCC 6803 and PCC 7002 [33]. Segregation of mutants was not obtained after multiple streaking on plates containing 50 μg/mL kanamycin. Segregation was only observed when the concentration increased to 100 μg/mL.

Prior to generating unmarked mutants, wild-type and the Δ*ctaC1D1E1* and Δ*ctaCII* marked mutants were cultured on plates with increasing concentrations of 5-FC (Figure S13). Wild-type cells grew well up to concentrations of 0.5 mg/mL but growth was poor at 1 mg/mL. Marked mutants did not grow, even on the lowest concentration of 0.05 mg/mL. This suggests that 5-FC is lethal to PCC 11901 cells expressing CodA.

Multiple methods of unmarking were trialed in two different laboratories with different alterations applied in each case. Following transformation of at least six different marked mutants targeting each of the three loci with the unmarking plasmids (Table S2) and incubation for four days in a shaking incubator, transformants were selected on plates with 0.25 mg/mL 5-FC added directly to the plate or to an agar layer added 24 h after the initial plating. No segregated unmarked colonies were obtained under any of these conditions at this stage. Colonies were then re-streaked on plates with increasing concentrations of 5-FC, up to 1 mg/mL. However, again no mutants were obtained with the expected unmarked profile (Figure 5C,F,I). Increasing the incubation time after transformation from four to six or eight days did not result in segregated unmarked colonies. Sequencing of one of these clones confirmed that there were no mutations in *coda*, or the regions upstream and downstream of this gene. All mutants contained the marked mutation or in some cases also the wild-type profile. This suggests that CodA is a poor negative selectable marker and unsuitable for generating unmarked PCC 11901 mutants.

## 4. Discussion

PCC 11901 has enormous potential as a chassis for biotechnology and as a model species to identify factors leading to high growth and biomass accumulation in photosynthetic organisms. The tools and strains developed in this study will aid these goals although our inability to develop a successful unmarking system is an issue that needs to be addressed. Currently, there are five to seven antibiotic resistance cassettes which have been successfully used in model cyanobacterial species [40]. This limits the number of chromosomal modifications possible, which may be inadequate for most biotechnology applications. An additional issue is potential release into the environment of organisms containing genes encoding resistance to antibiotics. A system was recently developed in PCC 7002 using the CRE-lox recombinase system [41]. This relies on integrating an antibiotic resistance cassette flanked by lox66 and lox71 recombinase sites into the chromosomal target site to generate a marked knockout. A second plasmid encoding the CRE recombinase is then integrated into the essential *rbcLXS* locus using a second antibiotic resistance marker. Expression of CRE leads to excision of the antibiotic resistance cassette, resulted in generation of an unmarked mutant. Finally, growing this strain in the absence of the second antibiotic results in the gene encoding CRE being removed from the chromosomal population. While this method does not result in production of scarless unmarked mutants or precise genetic engineering, unlike with the SacB method [34], strains can be repeatedly genetically manipulated. Therefore, this may be a useful method to attempt in the future. Regardless, if new negative selectable genes are discovered which can be used in bacteria, then these should be tested in PCC 11901 in future studies. CRISPR-based genome editing could be another alternative, which has been used to generate segregated mutants in 6803 [42].

Another drawback of PCC 11901 is the potentially higher polyploidy in this species compared to PCC 6803 or PCC 7002. For example, generation of segregated marked knockouts of *ctaC1D1E1* and *ctaCII* in PCC 6803 in our laboratory was achieved after two streaks on agar plates containing kanamycin at a concentration of 30 μg/mL [19]. However, in PCC 11901, more streaks on plates containing a higher concentration of kanamycin, up to 100 μg/mL, was required. Polyploidy needs to be determined in this species under a range of growth and nutrient conditions, as has previously been performed for PCC 6803 [43]. It is possible that growing PCC 11901 on low phosphate media may reduce polyploidy and make it easier and quicker to generate marked mutants, as has been shown in PCC 6803 [44]. This may render CRISPR-based genome editing approaches more viable in this species.

Directed evolution of PCC 11901 to overcome dependence on vitamin B_12_ resulted in strains that, following an initial lag, were able to grow in the absence of this component (Figure 4). This initial lag cold be due to a higher requirement for methionine (for protein synthesis) at the early stages of growth, as the B_12_-independent methionine synthases are known to be less catalytically efficient than their B_12_-dependent counterparts in *E. coli* [45]. The double TT=>AA mutations observed in the B_12_ind_5 strain (Figure S12), while not localized on either the predicted aptamer or the terminator region of the riboswitch [46], are sufficient to enable wild-type transformed with pTS012 (ReB_12_ind_5) to have a similar growth profile to that of the spontaneous mutant strain (Figure 4). It is somewhat surprising that the wild-type PCC 11901 strain was also able to, following a long lag, grow in the absence of vitamin B_12_. As previously speculated [7], the loss of *metE* functionality may have been a relatively recent event and, under laboratory conditions, the wild-type strain appears to have drifted from the original isolate, which was completely incapable of growth in the absence of vitamin B_12_. Genetic drift of wild-type strains under laboratory conditions is a well described phenomenon [47] and a thorough resequencing of the genome and plasmids may reveal other genome loci that have changed during laboratory cultivation. Nevertheless, this advantageous trait could be further exploited, as it reduces scale-up costs and introduction of further *metE* gene copies could improve growth in the early growth phase in the absence of vitamin B_12_.

Our data confirms that PCC 11901 outperforms all other model cyanobacterial species in terms of sustained growth, at least in small-scale laboratory experiments. However, growth of this species has not been tested at larger scale and in outdoor photobioreactors. Additionally, the optimal light intensity for growth has not been established. There are several factors identified in this study that could underlie the fast growth of this species. Photoinhibition was lowest in PCC 11901, although not significantly different from UTEX 2973 after 20 min (Figure 3D,E). This could be one factor underlying the fast growth of both species at low cell densities, when photoinhibition has the greatest impact. Photosynthetic rates, in addition to light use, were significantly higher in PCC 11901 compared to the other species (Figure 3A–C). This could be due to differences in the electron transport chain or more efficient light harvesting (Figure 2). Surprisingly, the photosynthetic rate of PCC 6803 was far lower compared to the other species and lower than we have observed in previous studies [34,35]. The negative effect on oxygen generation could be due to the conditions which these cells were grown under prior to oxygen measurements (i.e., 125 µmol photons m^−2^ s^−1^ with 5% CO_2_ bubbling), or that oxygen generation was impeded by the transition from a high CO_2_ environment to the ambient air level in the oxygen electrode.

Comparative genomics suggest that the PBS of PCC 11901 is smaller than the other species examined in this study. This may be advantageous in dense cultures, since it may reduce light absorption of cells at the surface, reducing photoinhibition, while allowing additional light to penetrate into the photobioreactor interior, thereby increasing productivity. This could be a major factor leading to the faster growth of PCC 11901 observed at higher cell densities. Many studies tried to attenuate PCC 6803 PBSs to achieve a similar outcome [34,48,49,50]. However, PBS attenuation has not always led to an increase in growth in dense cultures, most likely due to unintended consequences. These include differences in cell size [34,51], thylakoid membrane morphology [51,52], and the overabundance of other proteins, including many involved in photosynthesis, likely due to the excess protein biosynthesis capacity available following lower PBS protein component biosynthesis [53].

Metabolic pathways were largely conserved between PCC 6803 and PCC 11901, although carbon flux may differ greatly between the two species. Given that PCC 11901 displays its fast growth phenotype under high carbon dioxide conditions it is possible that Rubisco carboxylation rates may differ greatly between the species examined in this study. The carboxylation rate of rubisco from PCC 7002 is higher than that of *Synechococcus elongatus* PCC 6301 (13.4 vs. 11.6, respectively [1/s]) [54], a species which is almost identical to PCC 7942 [55]. Higher carboxylation rates would result in greater turnover of NADP^+^/NADPH and ADP/ATP, therefore increasing photosynthetic rates and limiting over-reduction of the electron transport chain. In-depth metabolomics studies and enzyme kinetics of PCC 11901 Rubisco would be required to resolve this.

## 5. Conclusions

This study further demonstrates that PCC 11901 is a strong candidate for cyanobacterial biotechnology when cells are cultured under high CO_2_ conditions. Further development of this species for biotechnology applications will be aided by the bioinformatics analysis and vitamin B_12_ auxotrophic strain provided in this study. While we were unable to generate unmarked knockouts using available negative selectable markers, this study suggests that future approaches to develop systems for repeated genetic manipulation should focus on CRISPR or recombinase-based approaches. Overall, this study lays the foundation for the use of PCC 11901 as a robust chassis for renewable biotechnological applications, paving the way for efficient photosynthetic recovery of industrial CO_2_ waste streams and toward carbon-efficient biomanufacturing.

## Data Availability

The data that support the findings of this study are available from the corresponding author upon reasonable request.

## Supplementary Materials

The following supporting information can be downloaded at: https://www.mdpi.com/article/10.3390/biom12070872/s1: Figure S1: Correlation between the Abs(680 nm)-Abs(750 nm) value and amounts of chlorophyll measured following methanol extraction. Figure S2: Plasmids pTS011 and pTS012. Figure S3: Schematic detailing the pathways involved in PCC 11901 central metabolism. Figure S4: Metabolism and degradation of nucleotide sugars and sugar osmolytes in PCC 11901. Figure S5: Metabolism of amino acids, cyanophycin, glutathione and iron-sulfur clusters in PCC 11901. Figure S6: Metabolism of nucleotides in PCC 11901. Figure S7: Metabolism of vitamins and cofactors in PCC 11901. Figure S8: Metabolism of membrane lipids, peptidoglycan and lipopolysaccharides in PCC 11901. Figure S9: Metabolism of isoprenoids, quinols and carotenoids in PCC 11901. Figure S10: Metabolism of chlorophyll, phycobilin and pseudocobalamin in PCC 11901. Figure S11: Proteins involved in metabolite transport and conversion of nitrogen, sulfur and phosphate-based compounds in PCC 11901. Figure S12: Sequencing of the *metE* upstream region of the PCC 11901 B12ind_5 strain. Figure S13: Growth of wild-type 11901 and the *ctaC1D1E1* and *ctaCII* marked mutants on plates with increasing concentrations of 5-FC. Table S1: Primers used in this study. Table S2: Plasmids used in this study. Table S3: Potential PCC 11910 homologues of PCC 6803 proteins involved in central metabolism. Table S4: Potential PCC 11910 homologues of PCC 6803 proteins involved in processes other than central metabolism, light harvesting and photosynthesis, and proteins of unknown function. Table S5: PCC 11910 proteins with no homologues in PCC 6803. Table S6: Potential PCC 11910 homologues of PCC 6803 proteins involved in electron transport and light harvesting. Reference cited in [56].
