# Supplementary material for: Development of a Biotechnology Platform for the Fast-Growing Cyanobacterium Synechococcus sp. PCC 11901
Source: Biomolecules. 2022 Jun 23;12(7):872. doi: 10.3390/biom12070872 (PMC9313322; doi:10.3390/biom12070872)
Supplement: Supplementary file 1 [file biomolecules-12-00872-s001.zip › Supplementary information_B.pdf]

Supplementary information

Figures S1 – S13.

Tables S1 – S6.

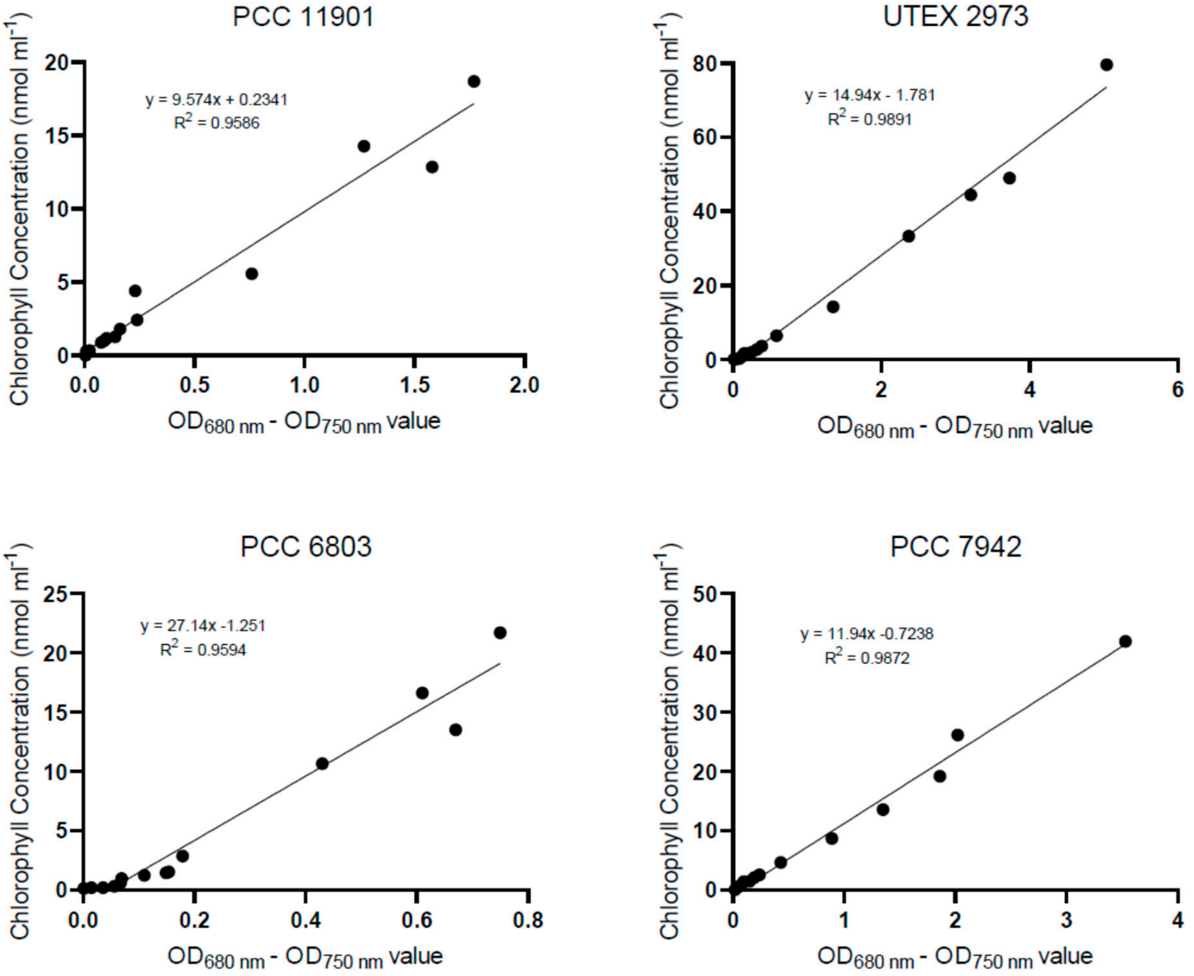

Figure S1: Correlation between the Abs(680nm)-Abs(750nm) value and amounts of chlorophyll measured following methanol extraction. Samples were measured at absorbance of 750nm and 680nm, followed by extraction with methanol to measure chlorophyll concentration. Amount of chlorophyll was correlated with absorbance (A_{680}-A_{750}). The regression line is shown. The slope of the regression line (R^2) was then calculated.

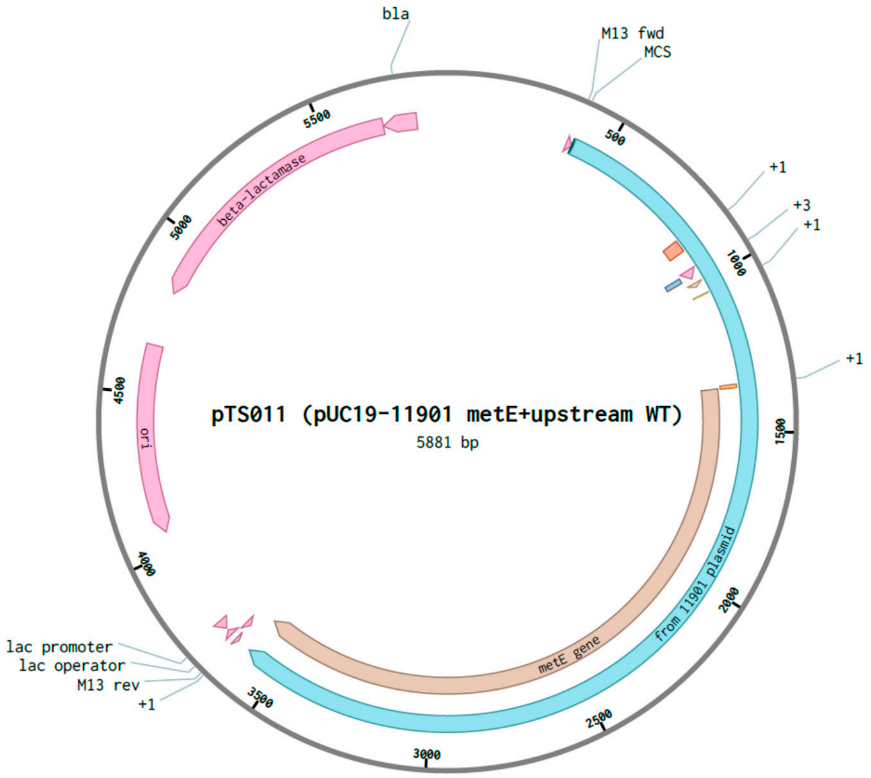

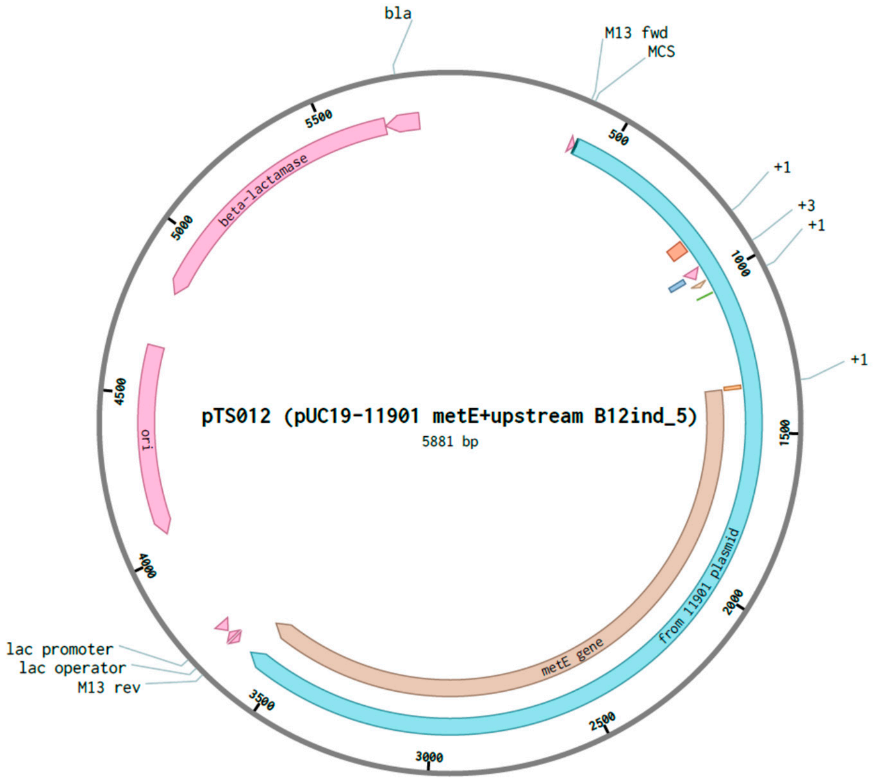

Figure S2: Plasmids pTS011 and pTS012. Plasmid pTS011 (*top*) was used to sequence the region 1000 bp upstream and 500 bp downstream of the WT *metE* gene start codon, for comparison with both the published sequence and that of the B12ind_5 strain (cloned in pTS012, *bottom*). Annotated GenBank files can be found in

pTS011: https://benchling.com/s/seq-t6xsILttV82C2VMouGmb?m=slm-pIdDqYWKUrCx2dqMOCLo

pTS012: https://benchling.com/s/seq-hnUCIuMPolpxaGYDBPQs?m=slm-e9O29Gmllg5hovbBXnhF

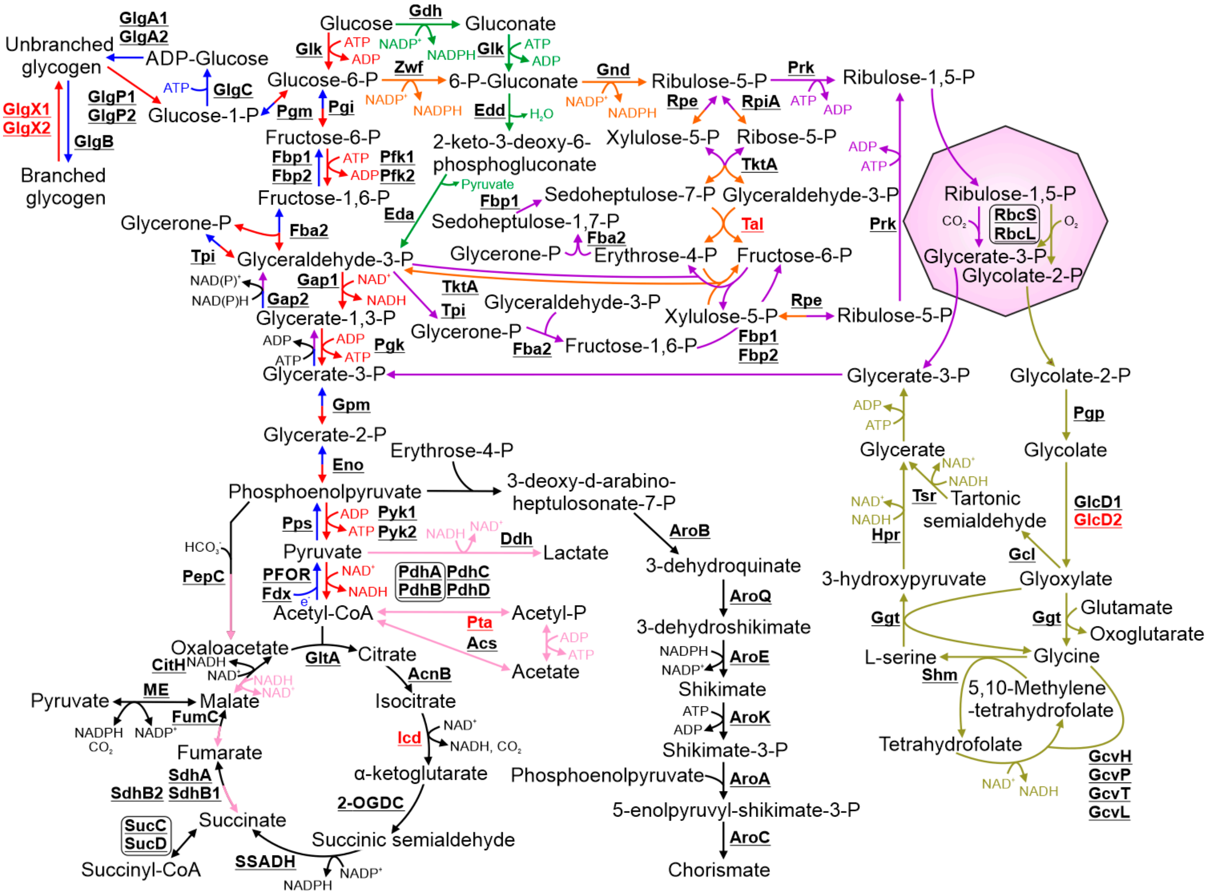

Figure S3: Schematic detailing the pathways involved in PCC 11901 central metabolism. Biosynthetic steps involved in glycolysis and gluconeogenesis are highlighted in red and blue respectively. Steps in the Entner-Doudoroff pathway are highlighted in green. Steps involved in the oxidative pentose phosphate pathway and the Calvin-Benson-Bassham cycle are highlighted in orange and purple, respectively. Fermentation pathways are highlighted in pink. Photorespiration pathways are highlighted in olive. Where enzymes catalyse reactions in two pathways, the arrows are split between their respective colours. The carboyxsome is represented as a purple octagon. Cofactors in each reaction are shown with the exception of protons, water, oxygen and inorganic phosphate. Proteins with low sequence similarity to PCC 6803 enzymes are highlighted in red.

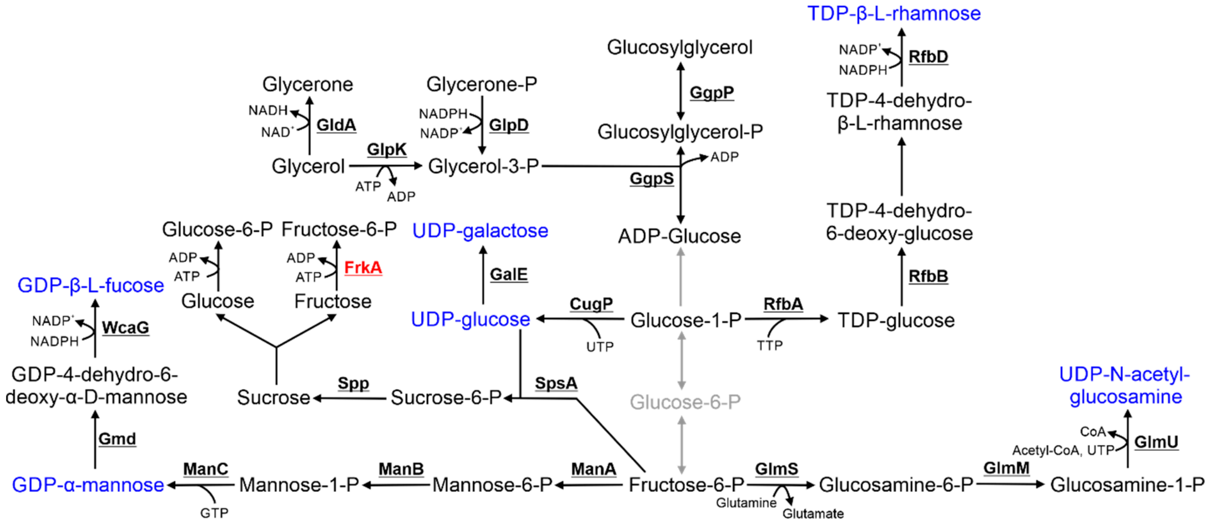

Figure S4: Metabolism and degradation of nucleotide sugars and sugar osmolytes in PCC 11901. Compounds highlighted in blue are substrates for lipopolysaccharide biosynthesis. Steps highlighted in grey are compounds and reactions involved in these pathways but detailed in figure 1. Cofactors in each reaction are shown with the exception of protons, water, oxygen and inorganic phosphate. Proteins with low sequence similarity to PCC 6803 enzymes are highlighted in red.

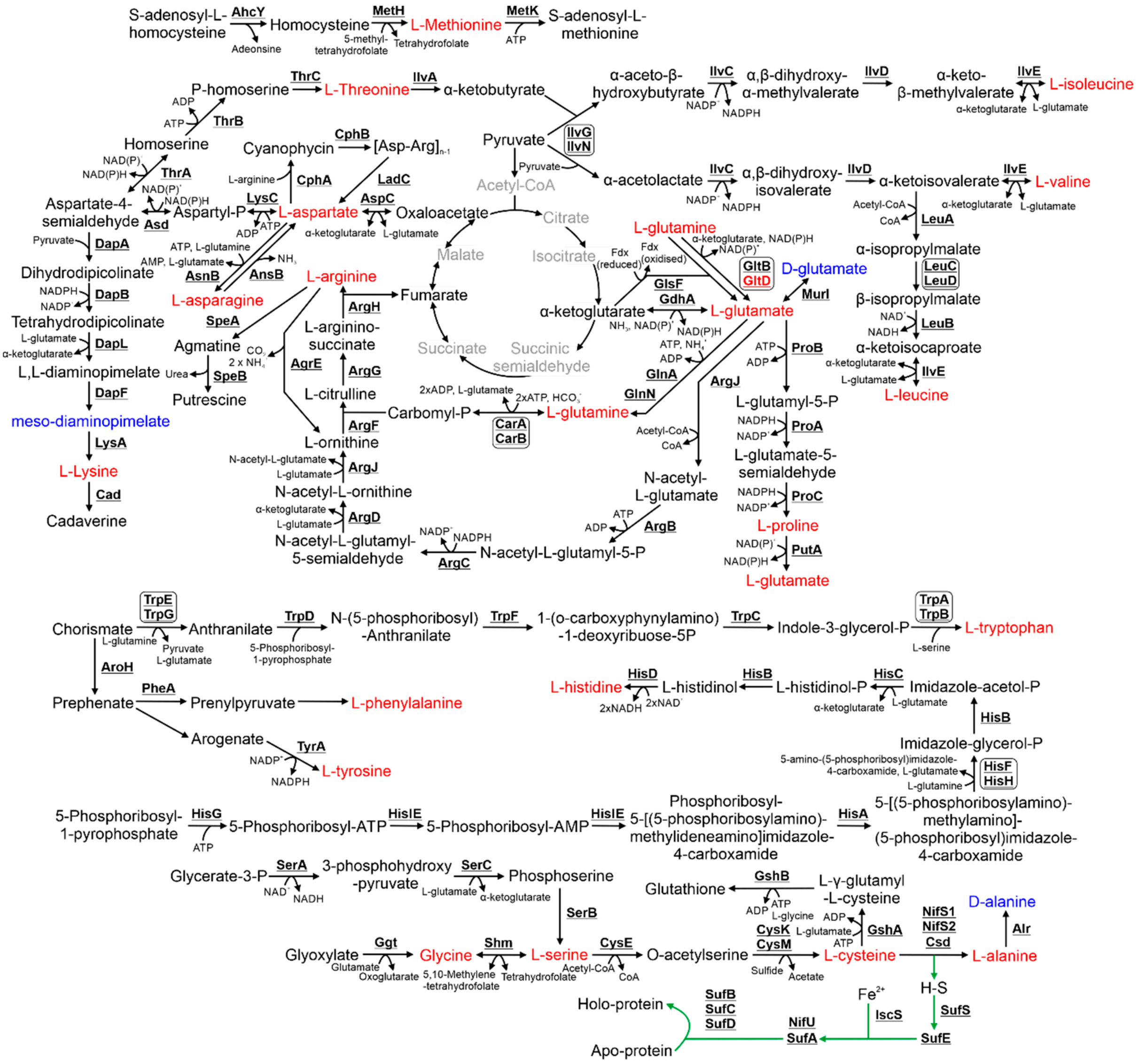

Figure S5: Metabolism of amino acids, cyanophycin, glutathione and iron-sulphur clusters in PCC 11901. The twenty L-amino acids are highlighted in red while amino acids incorporated into peptidoglycan are highlighted in blue. The iron-sulfur biosynthetic pathway is highlighted in green. Steps highlighted in grey are compounds and reactions involved in these pathways but detailed in figure 1. Cofactors in each reaction are shown with the exception of protons, water, oxygen and inorganic phosphate. Proteins with low sequence similarity to PCC 6803 enzymes are highlighted in red.

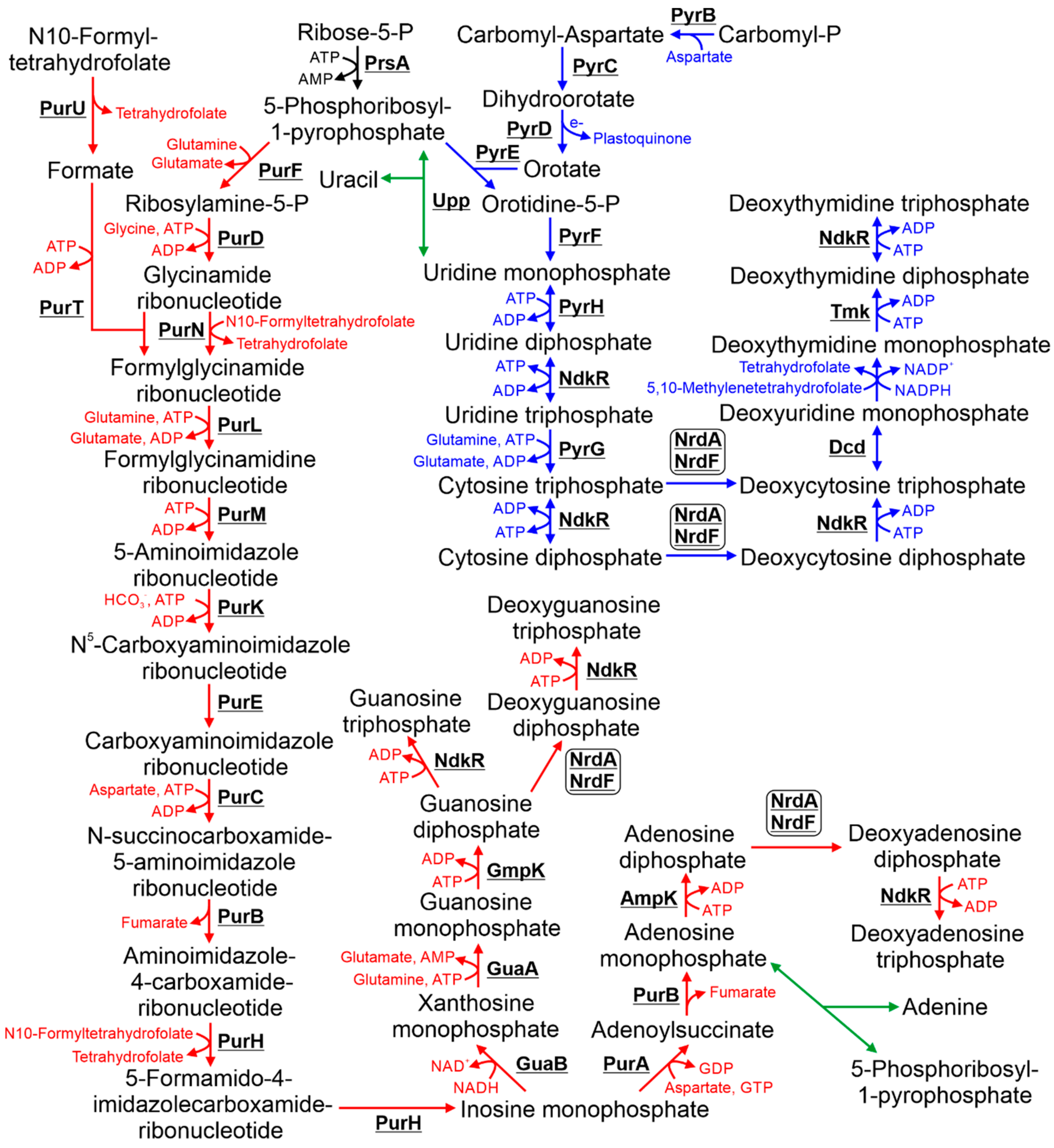

Figure S6: Metabolism of nucleotides in PCC 11901. The purine and pyrimidine biosynthesis pathways are highlighted in red and blue respectively. Possible nucleotide salvage pathways are highlighted in green. Cofactors in each reaction are shown with the exception of protons, water, oxygen and inorganic phosphate.

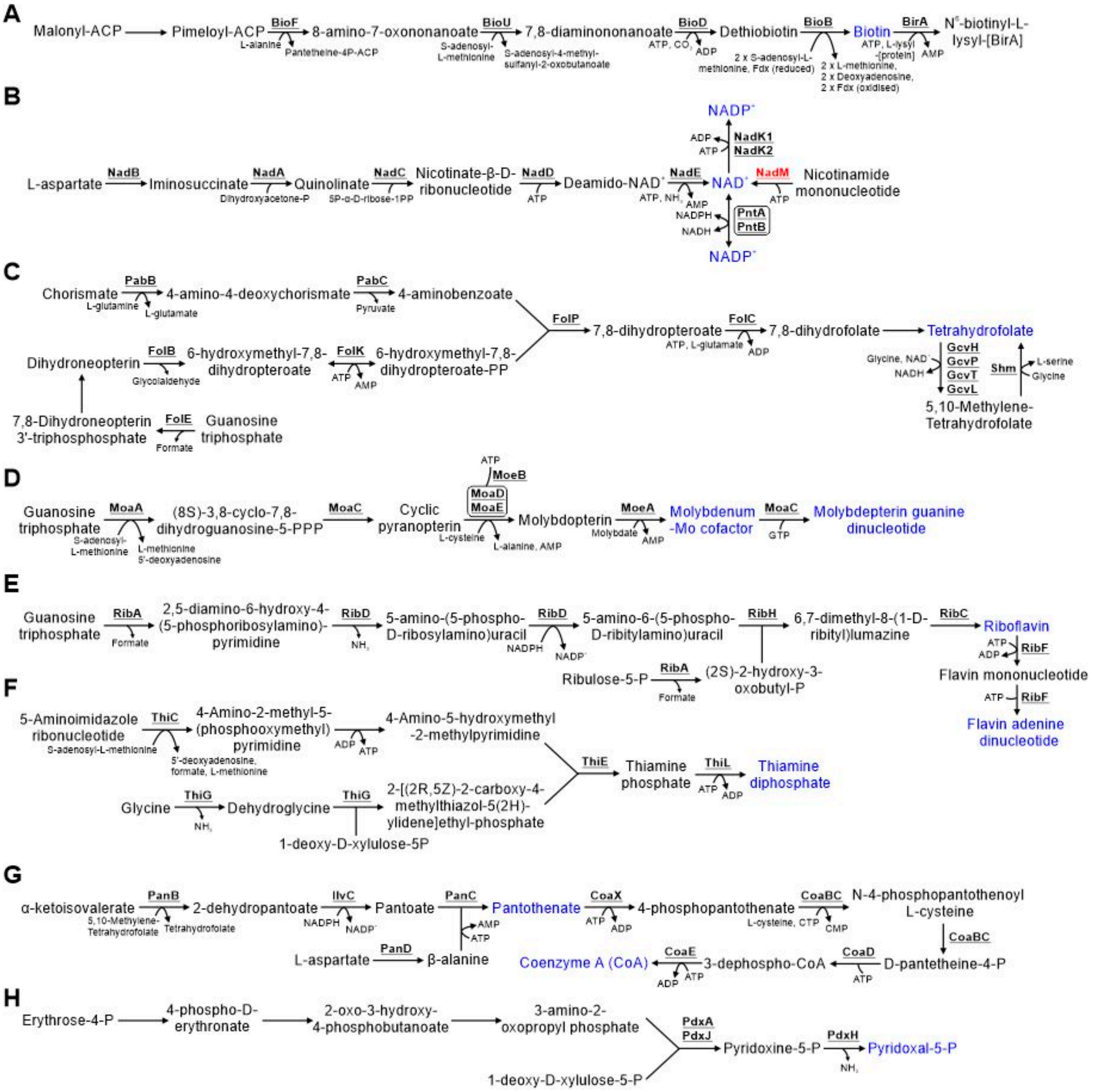

Figure S7: Metabolism of vitamins and cofactors in PCC 11901. Detailed are the pathways for biosynthesis of A) Biotin, B) NAD+ and NADP+, C) folate, D) molybdenum cofactors, E) riboflavin and FAD, F) thiamine, G) pantothenate and coenzyme A, H) pyridoxal-5P. Vitamins and cofactors are highlighted in blue. Cofactors in each reaction are shown with the exception of protons, water, oxygen and inorganic phosphate. Proteins with low sequence similarity to PCC 6803 enzymes are highlighted in red.

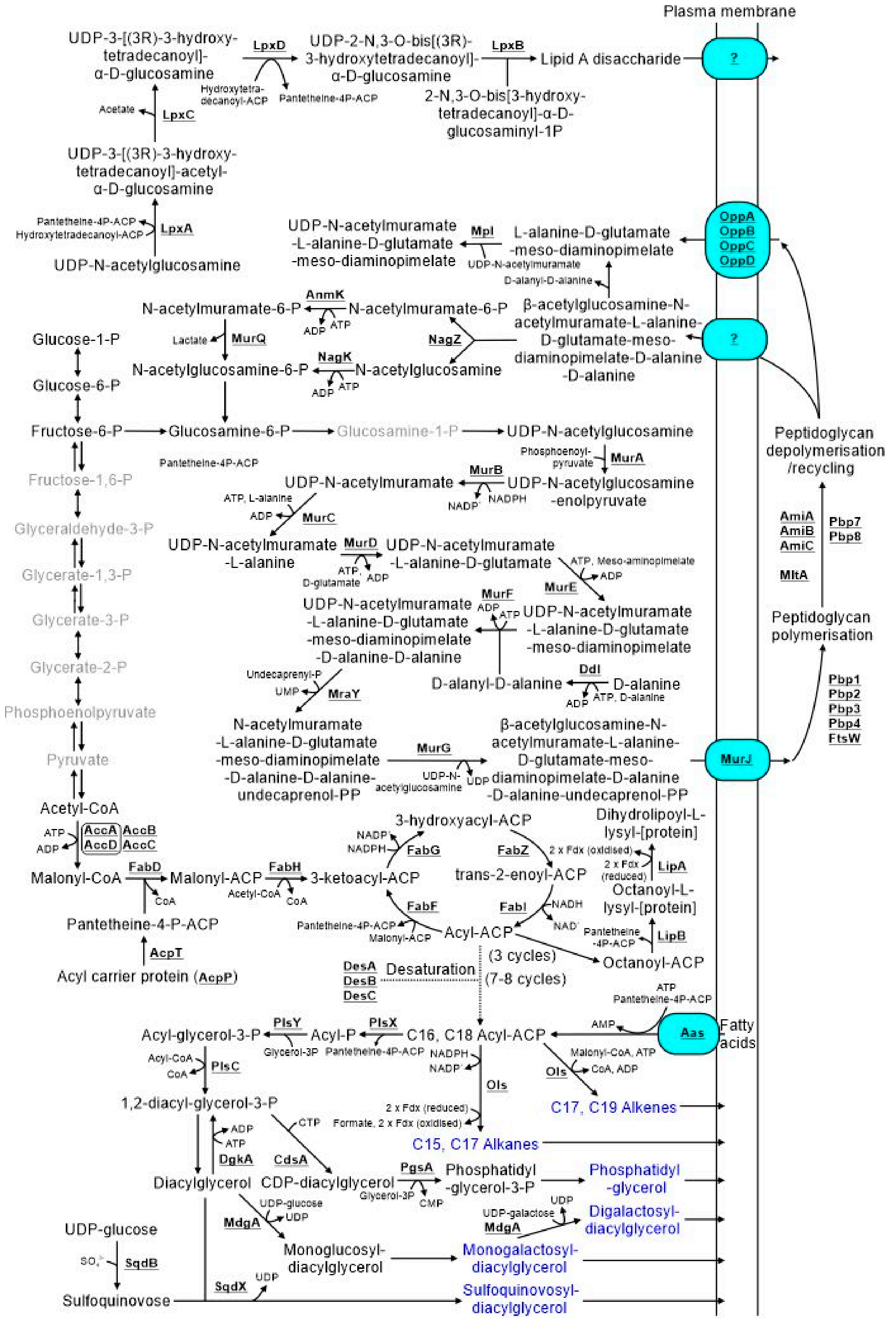

Figure S8: Metabolism of membrane lipids, peptidoglycan and lipopolysaccharides in PCC 11901. Membrane lipids are highlighted in blue. Steps highlighted in grey are compounds

and reactions involved in these pathways but detailed in figure 1. Cofactors in each reaction are shown with the exception of protons, water, oxygen and inorganic phosphate.

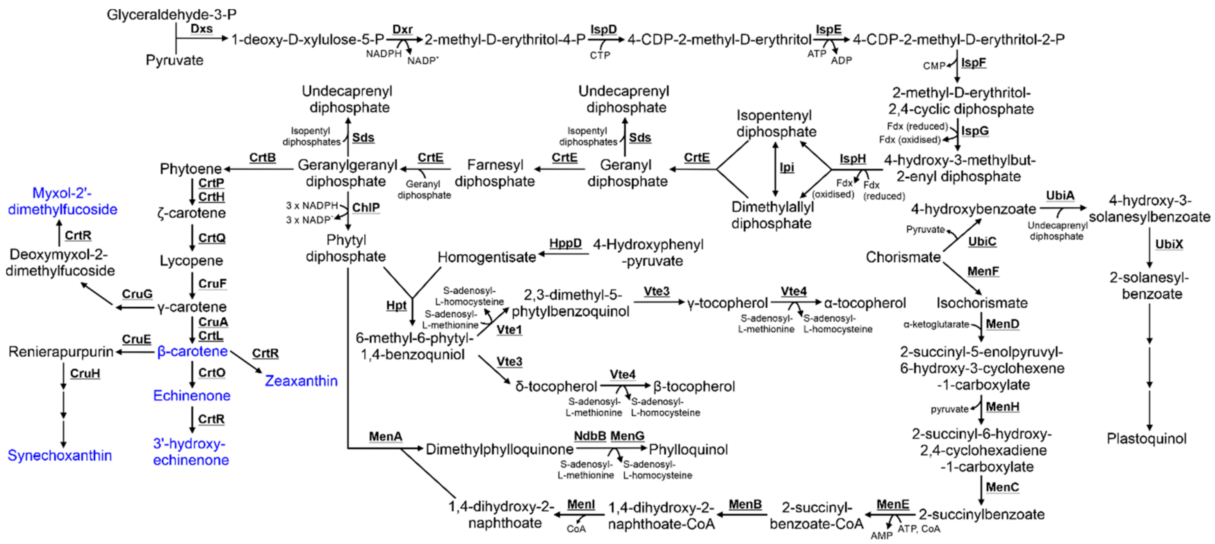

Figure S9: Metabolism of isoprenoids, quinols and carotenoids in PCC 11901. Carotenoids are highlighted in blue. Cofactors in each reaction are shown with the exception of protons, water, oxygen and inorganic phosphate.

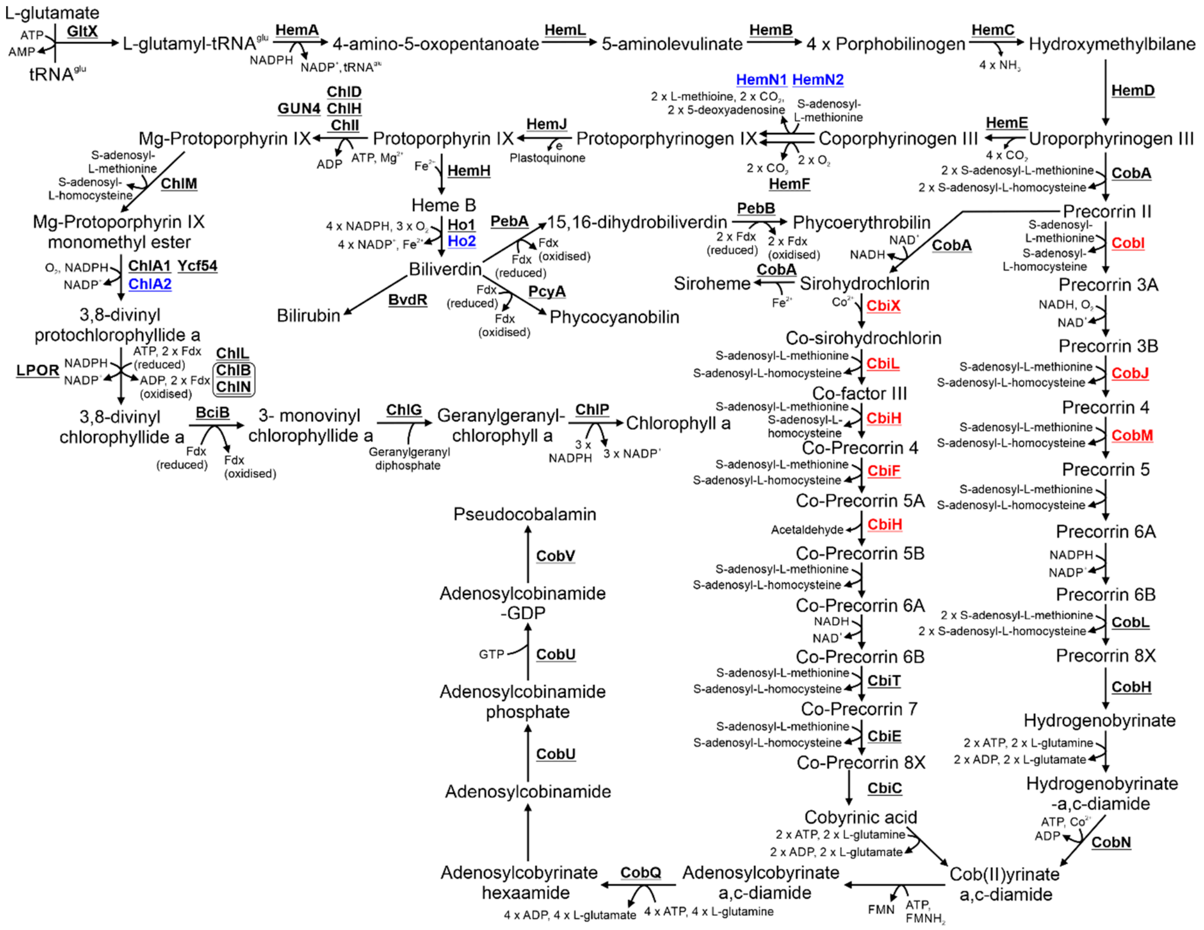

Figure S10: Metabolism of chlorophyll, phycobilin and pseudocobalamin in PCC 11901. Proteins involved in anaerobic or low oxygen environment enzymatic steps are highlighted in blue. Cofactors in each reaction are shown with the exception of protons, water and inorganic phosphate. Proteins with low sequence similarity to PCC 6803 enzymes are highlighted in red.

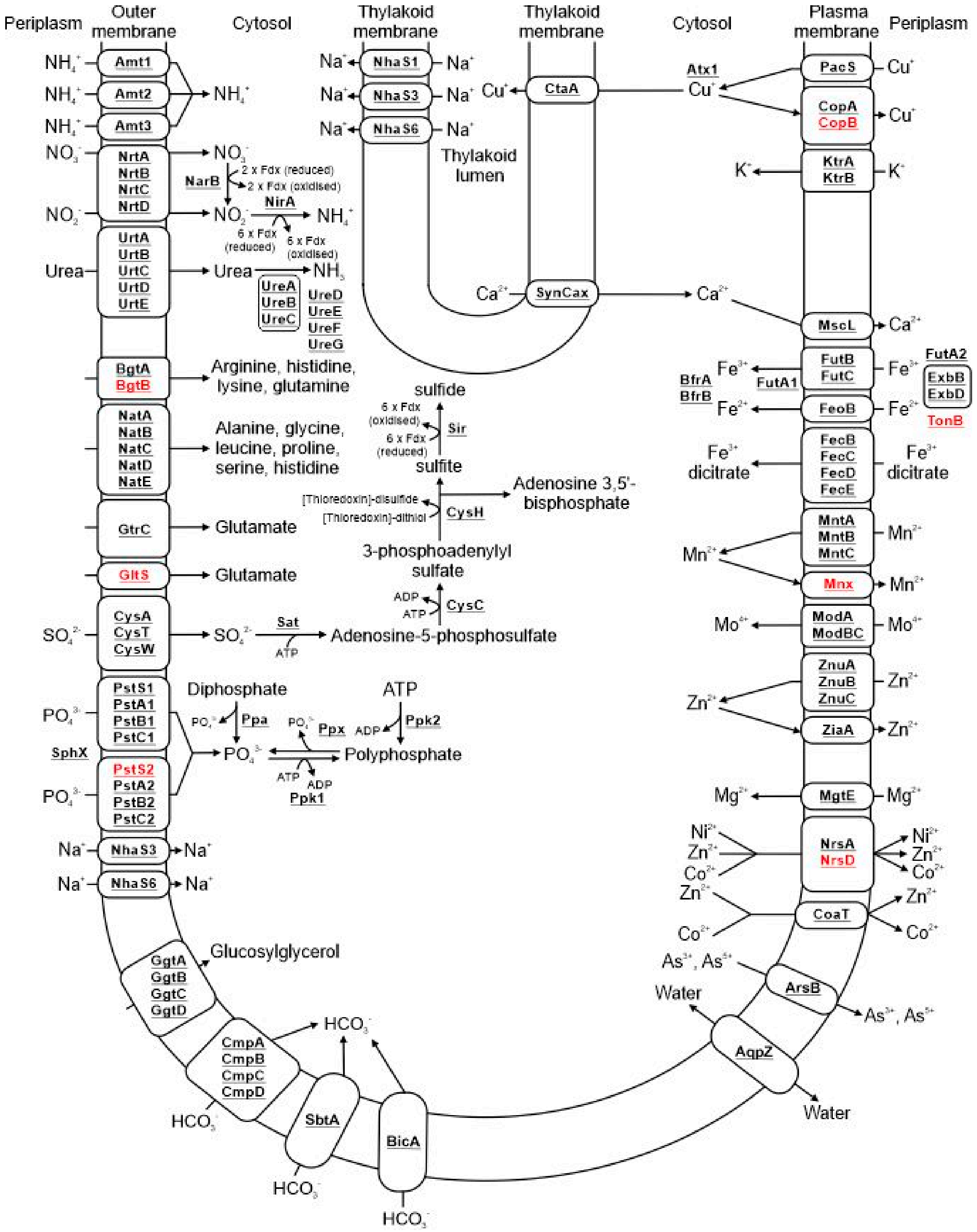

Figure S11: Proteins involved in metabolite transport and conversion of nitrogen, sulphur and phosphate based compounds in PCC 11901. Localisation of transporters in either the plasma or thylakoid membrane is detailed. Subunits in each complex may not all be membrane localised but soluble. Cofactors in each reaction are shown with the exception of protons, water, oxygen and inorganic phosphate. Proteins with low sequence similarity to PCC 6803 enzymes and transporter subunits are highlighted in red.

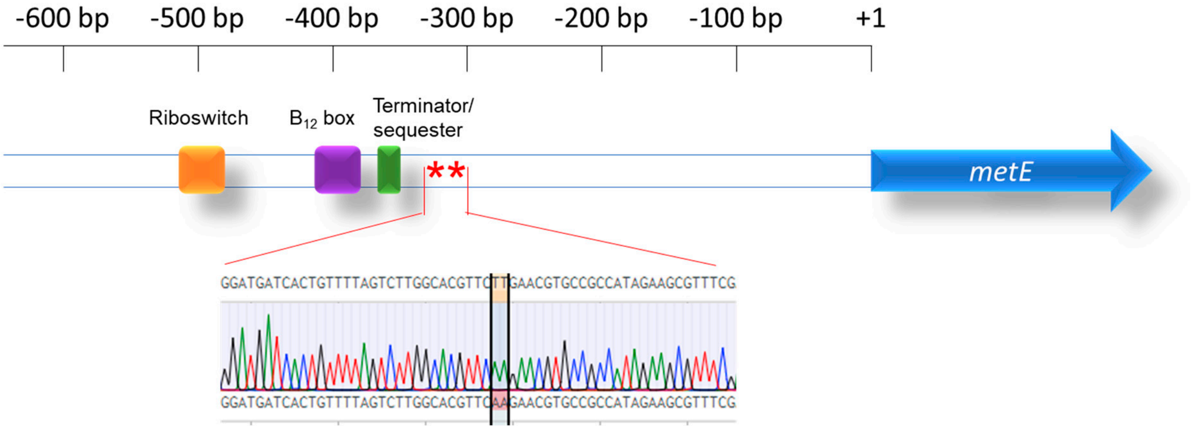

Figure S12: Sequencing of the *metE* upstream region of the PCC 11901 B12ind_5 strain. Riboswitch components, including B_{12} binding motif ("B_{12} box") and terminator/sequester, annotated as described by RibEx server. Insert shows sequencing chromatogram for the TT=>AA double mutation present in the B_{12}ind_5 strain and its alignment to the WT sequence.

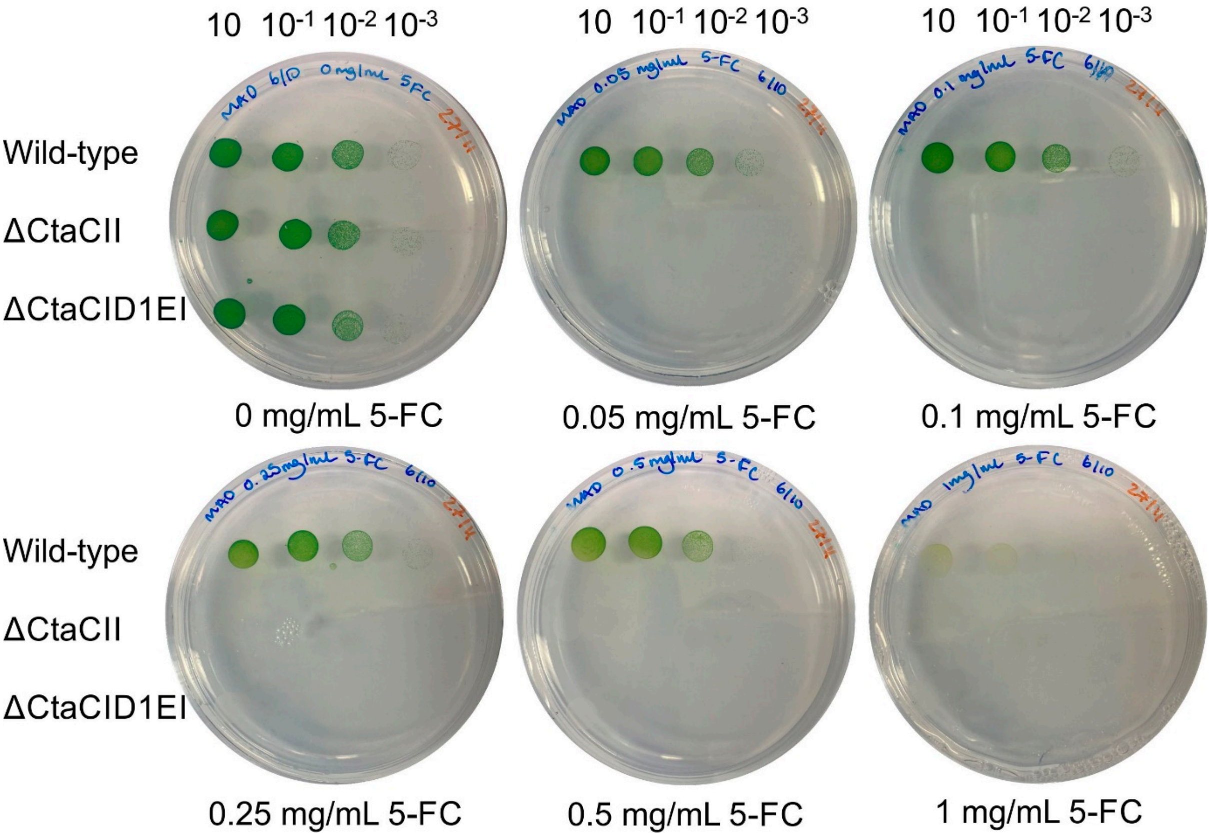

Figure S13: Growth of wild-type 11901 and the *ctaC1D1E1* and *ctaCII* marked mutants on plates with increasing concentrations of 5-FC. Dilutions are shown above each plate.

Table S1: Primers used in this study.

Primer	Sequence	Purpose
ICA_pUC19_11901metE_F	CACGACTTGGCAAGCTGCTTTTATTATCATGGTCATAGCTGTTTCCTG	Linearization of pUC19 backbone
ICA_pUC19_11901metE_R	TTGGATTTTTGATTTTTTGATCCCTAATTCACTGGCCGTCGTTTTAC	Linearization of pUC19 backbone
ICA_11901metE_pUC19_F	GTTGTAAAACGACGGCCAGTGAATTAGGGATCAAAAAATCAAAAATCCAATCAAG	Amplification of the 500 bp upstream and first 500 bp region of PCC 11901 metE for assembly to linearized pUC19
ICA_11901metE_pUC19R	CACAGGAAACAGCTATGACCATGATAATAAAAGCAGCTTGCCAAGTC	Amplification of the 500 bp upstream and first 500 bp region of PCC 11901 metE for assembly to linearized pUC19
metE_seq_1	AAAACCACTCAGGCATCACTG	Primer for Sanger sequencing
metE_seq_2	TCCTAGGCCAGCTCAAAGC	Primer for Sanger sequencing
metE_seq_3	TGCACGGGGAATTTGAGCG	Primer for Sanger sequencing
ARTO_LF	ATCGAGGTCTCAGGAGCGGAAACCAACCAGAGAAATT[illegible]	Generation of left flank
ARTO_LR	AGCTCGGTCTCTTCATGTAATCGCTCACAAACCATGACC[illegible]	Generation of left flank
ARTO_RF	TGCACGGTCTCAATAACATTGGTCACTAAAGCCTCTTG	Generation of right flank
ARTO_RR	GCTCAGGTCTCTAGCGTGTATGTCCACGGCACTTTG	Generation of right flank
COX_LF	ATCGAGGTCTCAGGAGCCTTTAGTGGTTGACGTGAA	Generation of left flank
COX_LR	AGCTCGGTCTCTTCATTCGCAAGTTCTGTGCGGACA	Generation of left flank
COX_RF	TGCACGGTCTCAATAAGATGAACCGCCGCATTGCCC	Generation of right flank
COX_RR	GCTCAGGTCTCTAGCGATCAGTGCCGTAATCCCGAA	Generation of right flank
desB_LF p1F	GTACGAAGACCTGGAGCTGACAGGTAAACTGCCGG	Generation and domestication of left flank
desB_LF p1R	GTACGAAGACTCTACTCGTTGAAACCCTTTCAAAT	Generation and domestication of left flank
desB_LR p2F	GTACGAAGACCGAGTAGACTGCCGCCC	Generation and domestication of left flank
desB_LR p2R	GTACGAAGACTCCATTAACAGTAGTCTGGGATGGCTGC	Generation and domestication of left flank
desB_RF	GTACGAAGACCTGCTTGAGCCATGCTCCGATTTGG	Generation of right flank
desB_RR	GTACGAAGACCTAGCGTTTTGGTAGGCGGTGGCG	Generation of right flank
COXfor	AGGCAATGTTCCCCTAGAGA	Confirmation of gene segregation
COXrev	ACCCCGTCAGCACATAGAAA	Confirmation of gene segregation
ARTOfor	CGAAGAGTAACATCACCGCC	Confirmation of gene segregation
ARTOrev	ATACGATGATTTGGCACGGC	Confirmation of gene segregation
desBfor	TGGGTTCAGTGGTAGTGGAC	Confirmation of gene segregation
desBrev	CCCAAGATCCCCGTTAAACC	Confirmation of gene segregation
Km_LF_QC	CAAGACGTTTCCCGTTGAAT	Verification of pUC19:ARTO-CodA-KanR and pUC19:COX-CodA-KanR
pUC19_LR_QC	GGTGATGACGGTGAAAACCT	
pUC19_RF_QC	GAGTCAGTGAGCGAGGAAGC	
Cassette_RR_QC	TTAGGCAGAAGACTGGGC	
FOR barcode	gtGGTCTCtATGaGAAGAGCACGGTAGCCTTNNNNNNNNNNNNNNNNNNNTGCCCAGTCTTCTGCCTAAG	Amplification of codA/kan cassette
REV insert	GTAGACATGAGCCGAAGGTC	Amplification of codA/kan cassette

Table S2: Plasmids used in this study.

Plasmid	Plasmid design	Purpose
pUC19: desB-CodA-Sp	pUC19 + 5´ flanking region of desB gene (1000 bp) + codA/SpR cassette (3488 bp) + 3´region of desB gene (1000 bp)	Marking plasmid: introducing codA-SpR into desB NS
pUC19: Unmark Linker	pUC19 + 59bp sequence	Serves as a blank/linker sequence to be inserted between the 5' flanking region and 3' flanking region of the desB gene for unmarking
pUC19: desB-Unmark Linker	pUC19 + 5´ flanking region of desB gene (1000 bp) + Unmark Linker (59 bp) + 3´region of desB gene (1000 bp)	Unmarking plasmid: introducing a short 60bp linker into the marked desB::codA-SpR
pTS011	pUC19 + 500 bp up-/downstream of WT *metE* start codon	Sequencing of WT PCC 11901 *metE* upstream region
pTS012	pUC19 + 500 bp up-/downstream of B12ind_5 *metE* start codon	Sequencing of B12ind_5 PCC 11901 *metE* upstream region; introducing B12ind_5 B12-independent phenotype to PCC 11901 WT
pUC19: ARTO-CodA-KanR	pUC19 + 5´ flanking region of ARTO gene (531 bp)+ codA/kanR cassette (2949 bp; amplified from vector pICH47732) + 3´region of ARTO gene (526 bp)	Generating marked mutant of ARTO in PCC 11901
pUC19: COX-CodA-KanR	pUC19 + 5´ flanking region of COX gene (745 bp)+ codA/kanR cassette (2949 bp; amplified from vector pICH47732) + 3´region of ARTO gene (658 bp)	Generating marked mutant of COX in PCC 11901
pUC19: ARTO-Unmark Linker	pUC19 + 5´ flanking region of ARTO gene (531 bp)+ + Unmark Linker (59 bp) + 3´region of ARTO gene (526 bp)	Generating unmarked mutant of ARTO in PCC 11901
pUC19: COX-Unmark Linker	pUC19 + 5´ flanking region of COX gene (745 bp)+ + Unmark Linker (59 bp) + 3´region of ARTO gene (658 bp)	Generating unmarked mutant of COX in PCC 11901

Table S3: Potential PCC 11910 homologues of PCC 6803 proteins involved in central metabolism. Proteins were identified in Mills *et al.* [1]. The Uniprot ID relates to the identification system used on the Uniprot database [2]. The Uniprot ID amino acid sequence stored within this database was then used during the BLASTp function. All Gene Products, Gene Name, Other Gene Names, Localisation, Molecular Weight (kDa) and No of TMH's are derived from Baers *et al.* (2019) [3]. Each blast hit shows the NCBI Accession, in addition to the Percentage Identity Score, the length of the alignment number of mismatches as well as the number of gaps within the alignment. The species start and end refers to the start and end of the alignment within each species. The E-value refers to the number of expected hits of a similar quality that could be found by chance, the lower the E-value, the less likely the match is down to chance. For this analysis, we have only included proteins with an E-value of 1 or less. The bit-score is a log2-scaled and normalised raw-score.

Table S4: Potential PCC 11910 homologues of PCC 6803 proteins involved in processes other than central metabolism, light harvesting and photosynthesis, and proteins of unknown function. Proteins were identified in Mills *et al.* [1]. The Uniprot ID relates to the identification system used on the Uniprot database [2]. The Uniprot ID amino acid sequence stored within this database was then used during the BLASTp function. All Gene Products, Gene Name, Other Gene Names, Localisation, Molecular Weight (kDa) and No of TMH's are derived from Baers *et al.* (2019) [3]. Each blast hit shows the NCBI Accession, in addition to the Percentage Identity Score, the length of the alignment number of mismatches as well as the number of gaps within the alignment. The species start and end refers to the start and end of the alignment within each species. The E-value refers to the number of expected hits of a similar quality that could be found by chance, the lower the E-value, the less likely the match is down to chance. For this analysis, we have only included proteins with an E-value of 1 or less. The bit-score is a log2-scaled and normalised raw-score.

Table S5: PCC 11910 proteins with no homologues in PCC 6803. Proteins identified as having no homology to PCC 6803's proteins where a BLAST E-value of 1 was used, and are therefore described as truly unique from the PCC 6803's proteome. The NCBI ID was taken from the PCC 11901's proteome release CP040360.1. The Uniprot ID relates to the identification system used on the Uniprot database [2]. The name, amino acid length and calculated molecular weight was taken from the NCBI Protein Database, as well as the date that the information was added.

Table S6: Potential PCC 11910 homologues of PCC 6803 proteins involved in electron transport and light harvesting. Proteins were identified in Mills *et al.* [1]. The Uniprot ID relates to the identification system used on the Uniprot database [2]. The Uniprot ID amino acid sequence stored within this database was then used during the BLASTp function. All Gene Products, Gene Name, Other Gene Names, Localisation, Molecular Weight (kDa) and No of TMH's are derived from Baers *et al.* (2019) [3]. Each blast hit shows the NCBI Accession, in addition to the Percentage Identity Score, the length of the alignment number of mismatches as well as the number of gaps within the alignment. The species start and end refers to the start and end of the alignment within each species. The E-value refers to the number of expected hits of a similar quality that could be found by chance, the lower the E-value, the less likely the match is down to chance. For this analysis, we have only included proteins with an E-value of 1 or less. The bit-score is a log2-scaled and normalised raw-score.

Supplementary References

1. Mills, L.A.; McCormick, A.J.; Lea-Smith, D.J. Current knowledge and recent advances in understanding metabolism of the model cyanobacterium Synechocystis sp. PCC 6803. *Biosci Rep* **2020**, *40*, BSR20193325.
2. UniProt, C. The Universal Protein Resource (UniProt) in 2010. *Nucleic Acids Res* **2010**, *38*, D142-148.
3. Baers, L.L.; Breckels, L.M.; Mills, L.A.; Gatto, L.; Deery, M.; Stevens, T.J.; Howe, C.J.; Lilley, K.S.; Lea-Smith, D.J. Proteome mapping of a cyanobacterium reveals distinct compartment organisation and cell-dispersed metabolism. *Plant Physiol* **2019**, *181*, 1721-1738.
